# An aptamer-drug conjugate for promising cancer therapy with comprehensive evaluation from rodents to non-human primates

**DOI:** 10.1038/s41392-025-02399-1

**Published:** 2025-09-24

**Authors:** Minhui Su, Yuan Liu, Hongxin Lin, Xiaoxing Wang, Danxia Ying, Lizhuan Zhang, Cai Yang, Mengyuan Jiang, Lujuan Xu, Xie Wang, Yang Sun, Haiyan Xu, Ziwen Zhang, Xiaojia Wang, Ting Fu, Sitao Xie, Jiaxuan He, Xiangsheng Liu, Weihong Tan

**Affiliations:** 1 Molecular Science and Biomedicine Laboratory (MBL), State Key Laboratory of Chemo/Biosensing and Chemometrics, College of Chemistry and Chemical Engineering, College of Biology, Aptamer Engineering Center of Hunan Province, Hunan University, Changsha, China; 2 Zhejiang Cancer Hospital, The Key Laboratory of Zhejiang Province for Basic and Clinical Application of Functional Nucleic Acids, Hangzhou Institute of Medicine (HIM), Chinese Academy of Sciences, Hangzhou, China; 3 Institute of Molecular Medicine (IMM), Renji Hospital, Shanghai Jiao Tong University School of Medicine, and College of Chemistry and Chemical Engineering, Shanghai Jiao Tong University, Shanghai, China

**Keywords:** Drug development, Drug delivery

## Abstract

Aptamers serve as unique targeting ligands, making aptamer-drug conjugates (ApDCs) an attractive strategy for targeted cancer therapy. This study performs a comprehensive evaluation from rodents to non-human primates (NHP) of a protein tyrosine kinase 7 (PTK7)-targeted ApDC (Sgc8c-M) made by conjugating the potent antimitotic agent monomethyl auristatin E (MMAE) to the classic PTK7 aptamer Sgc8c. Efficacy studies in various cancer types with PTK7 overexpression showed that Sgc8c-M effectively induces sustained tumor regression in cell line-derived and patient-derived xenografts, outperforming unconjugated MMAE, the chemotherapy drug paclitaxel, and a PTK7-targeted antibody-drug conjugate. Pharmacokinetic (PK) studies in mice revealed that Sgc8c-M leads to rapid accumulation and sustained MMAE levels in tumors, along with fast clearance from plasma and normal tissues. Further study in rats confirmed rapid clearance across most organs and revealed that over 75% of MMAE was excreted through urine and feces within 24 h. Toxicokinetic (TK) assessments indicated comparable systemic drug exposure without accumulation for repeated doses compared to single administration. Toxicity evaluations showed that the therapeutic dose with high efficacy was safe and that the toxicity resulting from extremely high doses could be reversibly controlled. Encouraged by these findings, we evaluated PK/TK profiles and safety of Sgc8c-M in cynomolgus monkeys. Similar to PK/TK profiles observed in rats, Sgc8c-M demonstrated good dose-dependent drug exposure. It was, moreover, well tolerated in monkeys with no obvious accumulation following multiple administrations. These findings highlight the potential of Sgc8c-M as an effective antitumor agent and provide useful insights for the clinical translation of emerging ApDCs.

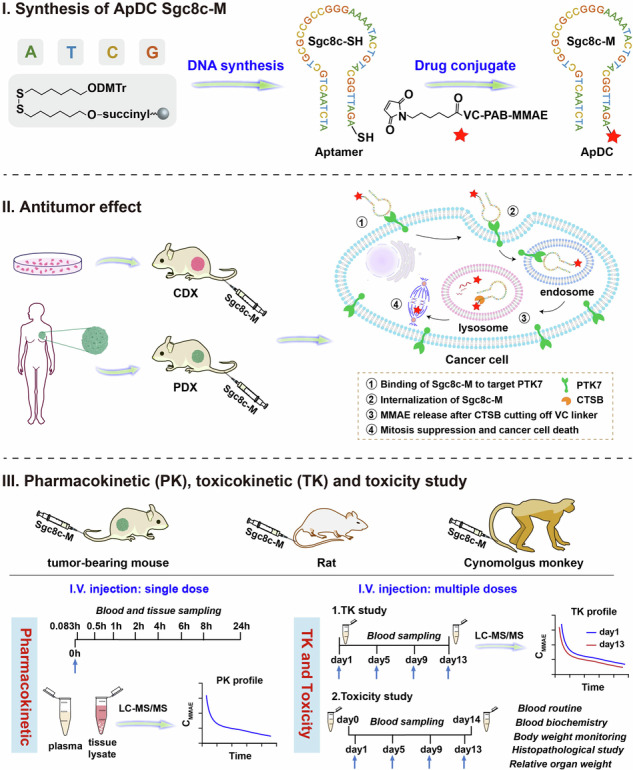

## Introduction

According to updated global cancer statistics from the International Agency for Research on Cancer (IARC), nearly 20 million new cancer cases and 9.7 million cancer-related deaths cases were recorded in 2022.^1^ Cancer has emerged as one of the foremost causes of disease-related mortality globally. Historically, chemotherapy and radiation therapy have been standard modalities in cancer treatment; however, these approaches invariably inflict damage to normal tissues, leading to serious adverse effects owing to their lack of tumor specificity. To address the poor specificity of traditional treatments, targeted drug delivery strategies have been developed, enabling the selective delivery of cytotoxic agents to tumor cells for precise killing.^2^ Targeted drug delivery substantially reduces systemic toxicity, while enhancing overall therapeutic efficacy. Antibody-drug conjugates (ADCs), which comprise monoclonal antibodies conjugated to cytotoxic drugs via chemical linkers, have emerged as one of the fastest-growing classes of therapeutics in oncology. Currently, fifteen ADCs have received approval from the U.S. Food and Drug Administration (FDA) with over 100 additional ADCs now in clinical trials.^3–5^ Following the development of ADCs, peptide-drug conjugates (PDCs) have also gained traction as next-generation targeted anticancer drugs, among which three, Lutathera, Pepaxto, and Pluvicto, successfully achieved market approval.^6,7^ Beyond antibodies and peptides, aptamers have also been explored as unique targeting ligands for the conjugation of cytotoxic drugs, leading to the construction of corresponding aptamer-drug conjugates (ApDCs).

Aptamers are short, single-stranded DNA or RNA molecules selected through systematic evolution of ligands by exponential enrichment (SELEX), a technology that selects aptamers able to bind specific targets with high specificity and affinity, just like antibodies.^8,9^ However, aptamers confer several distinct advantages, including facile synthesis and modification, low immunogenicity, chemical stability, and rapid tissue penetration, thereby positioning them as ideal targeting ligands for drug delivery in targeted cancer therapy.^10,11^ In numerous preclinical studies, ApDCs have exhibited promising tumor targeting and efficient antitumor efficacy as an emerging drug-targeting delivery strategy. In 2009, the first ApDC was constructed by conjugating the protein tyrosine kinase 7 (PTK7)-targeted aptamer Sgc8c with doxorubicin, demonstrating the homing capacity of aptamers conjugated with drugs to selectively kill cancer cells in vitro and validating the feasibility of aptamer-based targeted drug delivery.^12^ Since then, various ApDCs have been designed and synthesized for targeted cancer therapies. For example, in 2017, the Zhang group developed a nucleolin-targeted ApDC by conjugating aptamer NucA with paclitaxel, a clinical chemotherapy drug used in treating various cancers, including breast, ovarian, lung, and other cancers,^13^ and demonstrating its efficacy in a tumor model of ovarian cancer (OVCA).^14^ In 2020, our group reported the nucleolin-targeted AS1411-triptolide conjugate, which exhibited significant antitumor effects in the triple-negative breast cancer (TNBC) model.^15^ Furthermore, a PTK7-targeted Sgc8c-Combretastatin A4 conjugate was constructed by modularly engineered solid-phase synthesis and was employed to treat colorectal cancer (CRC) in vivo.^16^

Extensive studies indicate that PTK7 is overexpressed in various malignancies, including TNBC, non-small cell lung cancer (NSCLC), OVCA, CRC, pancreatic cancer, and acute lymphoblastic leukemia.^17–19^ The overexpression of PTK7 is frequently correlated with poor prognosis, tumor metastasis, and insufficient overall survival.^17,20,21^ Given the absence of catalytic activity, the development of small-molecule inhibitors targeting PTK7 presents a considerable challenge. Currently, a series of PTK7-targeted ADCs, such as cofetuzumab pelidotin^17^ and MTX-13,^22^ have shown promising antitumor efficacy and have progressed toward the clinical stage. These findings underscore the clinical translation potential of using PTK7 as a target to develop ApDC drugs.

However, no recent clinical development of the previously reported PTK7-targeted ApDCs has occurred owing, in part, to their moderate tumor inhibition potency.^15,16^ Moreover, most studies on ApDCs have, thus far, primarily concentrated on demonstrating efficacy, while often neglecting systematic preclinical studies of pharmacokinetics (PK) and toxicokinetics (TK), which are indispensable for the clinical translation of therapeutic agents.^23^ In 2023, our group performed the first systematic pharmacokinetics study of the radiolabeled PTK7 aptamer ^68^Ga-NOTA-Sgc8c in humans using total-body positron emission tomography (PET).^24^ This study provided preliminary data supporting human biosafety and metabolic patterns enabling the clinical translation of aptamers. However, the fate of ApDCs is still complicated by an assortment of factors, including proper dosage and the variety of different conjugated drugs. Therefore, we decided to move forward with the development of a PTK7-targeted ApDC with more robust potency and test it with a comprehensive preclinical pharmaceutical evaluation.

More specifically, we developed a PTK7-targeted ApDC, termed as Sgc8c-M, by choosing the classic aptamer Sgc8c and conjugating it with the potent auristatin microtubule inhibitor monomethyl auristatin E (MMAE), the most widely used toxic payload in ADCs, via cathepsin B (CTSB)-cleavable valine-citrulline-based linker. We then validated the strong targeting ability of Sgc8c-M both in vitro and in vivo. Sgc8c-M demonstrated superior antitumor efficacy when compared to unconjugated MMAE, the commonly used chemotherapy drug paclitaxel, or a PTK7-targeted ADC with the same MMAE payload across various cell line-derived xenografts (CDXs) and patient-derived xenografts (PDXs), including triple-negative breast cancer, pancreatic cancer, ovarian cancer, colorectal cancer, and non-small cell lung cancer. Additionally, we conducted a comprehensive preclinical PK and TK evaluation of Sgc8c-M in rodents and non-human primate cynomolgus monkeys. These studies allow us to better understand the properties of ApDCs and provide a critical research foundation for their future clinical translation.

## Results

### Synthesis and characterization of Sgc8c-M

**Fig. 1:**
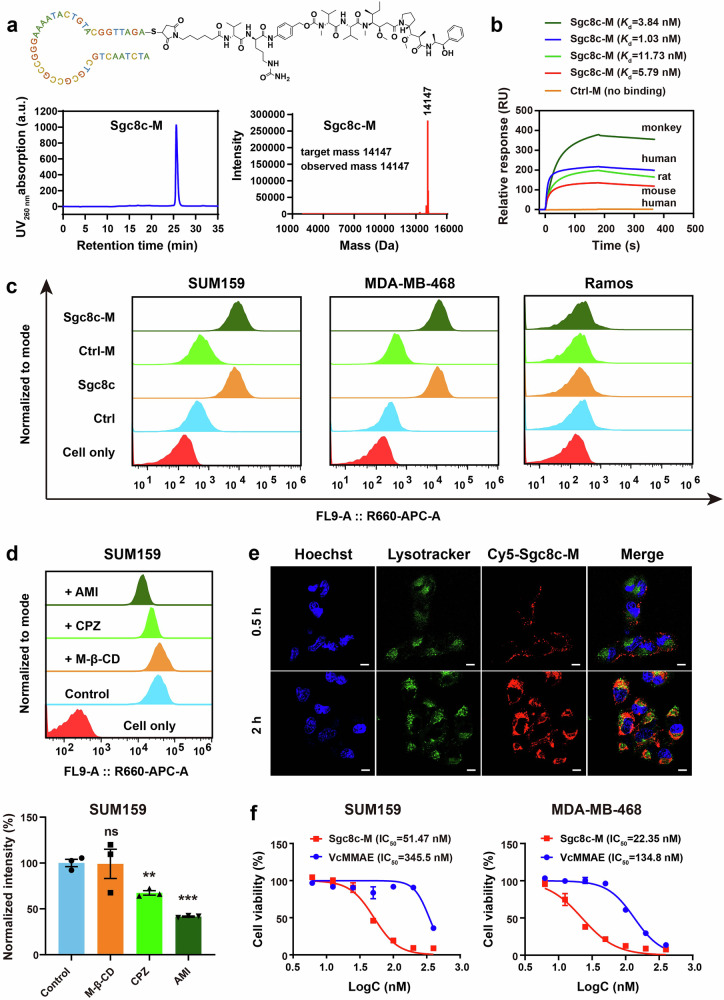
Characterization of Sgc8c-M in synthesis, targeting, internalization, and cytotoxicity in vitro. **a** Chemical structure, HPLC, and mass spectrum of Sgc8c-M. **b** Surface plasmon resonance (SPR) analysis of Sgc8c-M for binding to recombinant PTK7 proteins from humans, mice, rats, and cynomolgus monkeys at 400 nM. **c** Flow cytometry analysis of Cy5-labeled Ctrl, Sgc8c, Ctrl-M, and Sgc8c-M (250 nM) for binding to PTK7-positive SUM159 and MDA-MB-468 cells, as well as PTK7-negative Ramos cells. **d** Flow cytometry analysis of the internalization and endocytic pathway of Cy5-Sgc8c-M in SUM159 cells. The endocytic pathway was determined by measuring the uptake upon the application of various inhibitors. M-β-CD (0.2 mM), CPZ (25 μM), and AMI (100 μM) were preincubated with cells for 30 min at 37 °C; then 400 nM Cy5-Sgc8c-M were added and incubation continued for another 2 h (*n* = 3). Unpaired t test was used in comparison to control, **P < 0.01, ***P < 0.001, ns, not significant. **e** Confocal microscopy analysis of Cy5-Sgc8c-M (400 nM) for internalization in SUM159 cells at 0.5 h and 2 h. Scale bars, 10 μm. **f** Cytotoxicity of VcMMAE and Sgc8c-M in SUM159 and MDA-MB-468 cells after a 72-h incubation (*n* = 3). Data are presented as mean ± SEM

To construct ApDC Sgc8c-M, we employed a one-step Michael addition reaction between the 3’-thiol-modified aptamer Sgc8c-SH and the maleimide-modified drug MC-VC-PAB-MMAE (VcMMAE) (Supplementary Fig. 1a). The successful synthesis and purification of Sgc8c-M were confirmed using high-performance liquid chromatography (HPLC) and mass spectrometry (MS). HPLC and MS results indicated that the purity of Sgc8c-M exceeded 99% with exact molecular weight corresponding to the target mass of 14,147 Da (Fig. 1a). Moreover, negative control Ctrl-M (constructed using the inverted sequence of Sgc8c conjugated to MMAE), Cy5-labeled Sgc8c-M, and Cy5-labeled Ctrl-M were all synthesized under identical conditions (Supplementary Fig. 1b). This method demonstrated high efficiency, achieving a yield exceeding 90% within a 3-h reaction period, and is suitable for drug conjugation with various sequences. Overall, this approach offers a general and simple methodology for synthesizing ApDCs, which is pivotal for translational development.

### Targeting, internalization, and cytotoxicity of Sgc8c-M in vitro

Initially, we investigated the targeting of Sgc8c-M at both protein and cellular levels in vitro. At the protein level, Sgc8c demonstrated effective binding to PTK7 recombinant proteins from mouse, rat, cynomolgus monkey, and human with comparable equilibrium dissociation constants (*K*_d_) observed among these species (Supplementary Fig. 2). After MMAE conjugation, Sgc8c-M retained robust binding affinity for PTK7 proteins across different species with *K*_d_ values similar to those of unconjugated aptamer Sgc8c (Fig. 1b). In contrast, Ctrl-M showed no binding to human PTK7 recombinant proteins. The PTK7-targeted ADC h6M24-vc0101 previously showed no cross-reactivity with mouse and rat PTK7.^17,22^ These findings indicated that Sgc8c-M may possess consistent therapeutic effectiveness across species, which is a distinct advantage of ApDCs compared to some ADCs. At the cellular level, the aptamer was labeled with Cy5 dye enabling the study of its binding with cells by flow cytometry. Focusing on the PTK7-positive TNBC cell lines SUM159 and MDA-MB-468, we observed that both Cy5-Sgc8c and Cy5-Sgc8c-M exhibited significantly higher binding affinity for these cells compared to the controls, Cy5-Ctrl and Cy5-Ctrl-M (Fig. 1c and Supplementary Fig. 3a). Notably, Cy5-Sgc8c and Cy5-Sgc8c-M demonstrated no substantial binding to PTK7-negative Ramos cells. These results indicate that the affinity and specificity of Sgc8c encounter no interference as a result of MMAE conjugation.

Subsequently, we employed flow cytometry to examine the internalization and endocytic pathway of Cy5-Sgc8c-M in cells. We used three inhibitors of classical internalization pathways: amiloride hydrochloride (AMI) for the macropinocytosis pathway, chlorpromazine hydrochloride (CPZ) for the clathrin-mediated pathway, and methyl-β-cyclodextrin (M-β-CD) for the caveolae-mediated pathway. The working concentrations of these inhibitors were confirmed to be non-cytotoxic to cells (Supplementary Fig. 3b). In SUM159 cells, the internalization of Cy5-Sgc8c-M was significantly inhibited by both AMI and CPZ, with AMI demonstrating a more pronounced inhibitory effect (Fig. 1d). These results suggest that Cy5-Sgc8c-M is internalized into SUM159 cells through both macropinocytosis and clathrin-mediated endocytosis. Clathrin-mediated endocytosis is a type of the receptor-mediated endocytosis, indicating that Cy5-Sgc8c-M specifically recognizes and binds to PTK7 on the cell membrane, subsequently internalizing into SUM159 cells via this pathway. Regarding macropinocytosis, previous studies have demonstrated it can be stimulated by receptor tyrosine kinase EGFR.^25^ Notably, PTK7 has been shown to promote TNBC metastasis and progression through the EGFR/Akt signaling pathway,^21^ suggesting a plausible link between PTK7 and macropinocytosis. The endocytosis pathway of Sgc8c-M could be further validated in the future by siRNA mediated knockdown or genetic depletion of key endocytosis regulators. Interestingly, in MDA-MB-468 cells, the internalization of Cy5-Sgc8c-M was only inhibited by the macropinocytosis inhibitor AMI (Supplementary Fig. 3c). To visualize the internalization process, we performed confocal microscopy and observed that Cy5-Sgc8c-M could rapidly enter the lysosomes of SUM159 cells. After 0.5 h, Cy5-Sgc8c-M was primarily localized on the cell membrane, whereas at the 2-h mark, most Cy5-Sgc8c-M colocalized with lysosomes (Fig. 1e). Finally, we assessed the cytotoxicity of Sgc8c-M in both SUM159 and MDA-MB-468 cells, revealing that Sgc8c-M exhibited greater toxicity compared to unconjugated VcMMAE (Fig. 1f). Upon conjugation with the aptamer, we speculated that (1) VcMMAE is better able to bind to target cells and internalize into lysosomes and (2) the release of MMAE to exert its cell-killing effects was assisted by a specific linker molecule that breaks down once the conjugate reaches its target cells, allowing the drug to be released inside the cell. We also evaluated Sgc8c-M cytotoxicity in human normal ovarian epithelial cells IOSE80 and PTK7-negative cancer cells A549. The IC_50_ values for these cells were approximately 6-fold higher than that observed in PTK7-positive SUM159 cancer cells (Supplementary Fig. 4), demonstrating Sgc8c-M can kill PTK7-positive tumor cells with high specificity.

### Potent antitumor effect in TNBC models

PTK7 is highly expressed in TNBC and is associated with poor prognosis.^21^ Some studies have addressed this poor outcome by investigating PTK7 ADC to treat TNBC.^17,22,26^ To extend these studies, we first tested the therapeutic potential of Sgc8c-M on TNBC. To verify the in vivo targeting capability of Sgc8c-M, we employed in vivo fluorescence imaging of Cy5-Sgc8c-M in TNBC MDA-MB-468 tumor-bearing mice (Supplementary Fig. 5). Results showed that Cy5-Sgc8c-M exhibited significantly stronger fluorescence at tumor sites compared to Cy5-Ctrl-M at 90 and 120 min post-injection (Supplementary Fig. 5a). Following sacrifice at 2 h, ex vivo imaging revealed increased accumulation of Cy5-Sgc8c-M in tumors relative to that of Cy5-Ctrl-M (Supplementary Fig. 5c). Quantitative fluorescence analysis indicated that the concentration of Cy5-Sgc8c-M in tumors was more than 2-fold that of Cy5-Ctrl-M (*P* < 0.05), but with no significant differences observed in normal organs between the two treatment groups (Supplementary Fig. 5d). These findings highlight the excellent in vivo targeting of Sgc8c-M, demonstrating its specific enrichment in PTK7-overexpressing tumors. To clarify the targeting mechanism of Sgc8c-M, we further investigated the colocalization of Sgc8c-M and PTK7 protein by immunofluorescence imaging in another TNBC SUM159 models. Cy5-Sgc8c-M demonstrated obvious colocalization with PTK7 proteins in both cells and xenograft tumor sections (Supplementary Fig. 6). These findings provide direct visual evidence that Sgc8c-M can specifically target cancer cells with PTK7 overexpression in vitro and in vivo.

**Fig. 2:**
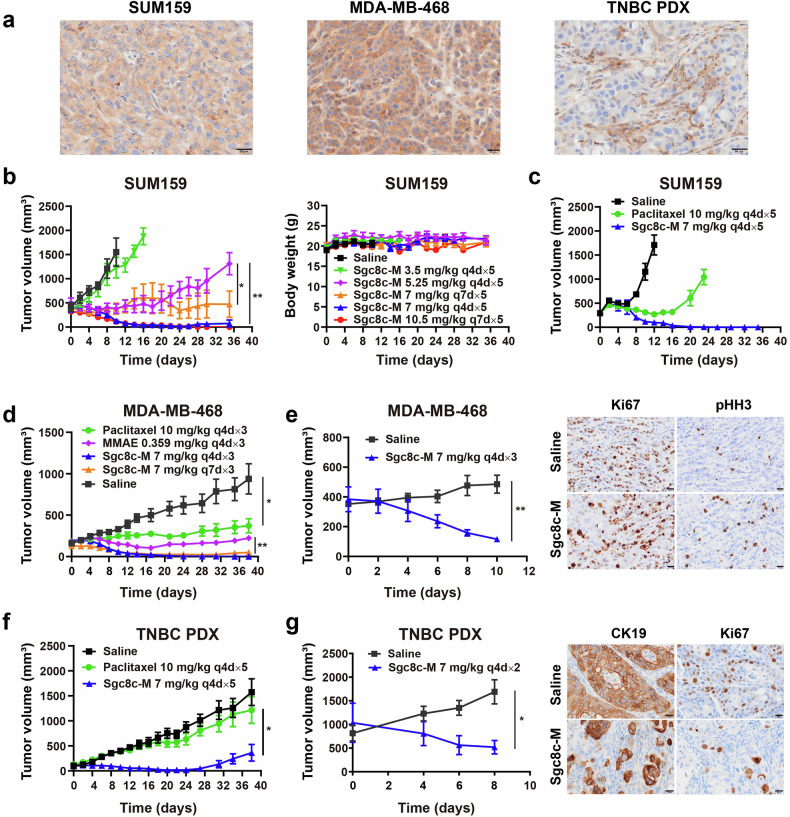
In vivo antitumor effect of Sgc8c-M in TNBC. **a** IHC characterization of PTK7 expression on tumor tissues of SUM159, MDA-MB-468, and TNBC PDX models. Scale bars, 20 μm. **b** Tumor volume and body weight of SUM159 tumor-bearing mice from a dose screening trial of Sgc8c-M (*n* = 4, one-way ANOVA). **c**, **d**, **f** Tumor growth curves for SUM159 (*n* = 4), MDA-MB-468 (*n* = 4, unpaired t test), and TNBC PDX (*n* = 5, unpaired t test) tumor-bearing mice after multiple dosing of 7 mg/kg Sgc8c-M (intravenous injection, i.v.), 10 mg/kg paclitaxel (intraperitoneal injection, i.p.), or 0.359 mg/kg MMAE (i.v.). **e**, **g** Tumor-killing effect study of Sgc8c-M in MDA-MB-468 (*n* = 3, unpaired t test) and TNBC PDX (*n* = 3, unpaired t test). Mice were given 7 mg/kg Sgc8c-M intravenously from day 0, and immunohistochemical characterization of CK19, Ki67, and pHH3 was performed at 48–96 h (48 h for MDA-MB-468, 96 h for TNBC PDX) at the end of administration. Scale bars, 20 μm. Data are presented as mean ± SEM. *P < 0.05, **P < 0.01

To optimize the dosing regimen for Sgc8c-M, we evaluated various administration doses and frequency in the TNBC SUM159 xenograft model. As shown in Fig. 2b, tumor growth curves indicated that a regimen of 3.5 mg/kg administered every four days for five cycles (q4d × 5) was insufficient to inhibit tumor growth in this model. However, increasing the dose to 5.25 mg/kg q4d × 5 and 7 mg/kg q7d × 5 significantly impeded tumor progression. Notably, the regimens of 7 mg/kg q4d × 5 and 10.5 mg/kg q7d × 5 exhibited robust antitumor effects, inducing sustained tumor regression. Body weight measurements indicated that mice tolerated all five dosing regimens very well without obvious weight loss. Based on these findings, 7 mg/kg q4d × 5 was selected as the optimal regimen for subsequent antitumor evaluations. We further compared the efficacy of Sgc8c-M against paclitaxel. In the SUM159 xenograft model, 10 mg/kg paclitaxel partially reduced tumor growth relative to saline controls, but the 7 mg/kg Sgc8c-M treatment resulted in nearly complete tumor regression (Fig. 2c). We further assessed the antitumor efficacy of Sgc8c-M in a TNBC MDA-MB-468 xenograft model, also characterized by high PTK7 expression (Fig. 2a). Unconjugated free MMAE was highly potent in vitro (Supplementary Fig. 3d). However, at equivalent doses, Sgc8c-M demonstrated superior tumor growth inhibition (TGI) compared to free MMAE (TGI: MMAE, 76% vs. Sgc8c-M q4d×3, 100%), indicating that conjugation with the aptamer significantly enhances the antitumor potency of MMAE in vivo (Fig. 2d). To our surprise, the 7 mg/kg q7d × 3 (TGI: 95%) and 7 mg/kg q4d × 3 regimens also exhibited comparable antitumor effects in the MDA-MB-468 model, both achieving sustained tumor regression. This may suggest that the MDA-MB-468 model is more sensitive to Sgc8c-M in comparison to the SUM159 model.

PDX models preserve the histopathological, molecular, and genetic characteristics of primary tumors, providing a more accurate platform for predicting clinical drug efficacy.^27,28^ Thus, alongside the aforementioned cell line-derived xenograft (CDX) models, we further assessed the antitumor efficacy of Sgc8c-M using a TNBC PDX model. As depicted in Fig. 2f, paclitaxel treatment failed to significantly inhibit tumor growth in the TNBC PDX model compared to saline controls. In contrast, Sgc8c-M demonstrated substantial antitumor efficacy with a period of complete tumor regression, although recurrence happened in the late stage.

Encouragingly, Sgc8c-M also demonstrated efficacy in inhibiting the growth of larger tumors. As illustrated in Fig. 2e, g, significant tumor growth inhibition was observed after administering Sgc8c-M for 2–3 doses, even when tumor volumes reached approximately 500 mm^3^. To further elucidate the tumor-killing effect of Sgc8c-M, we performed immunohistochemical (IHC) characterization of cytokeratin 19 (CK19, epithelial cytoskeleton marker), Ki67 (proliferation marker), and phosphohistone H3 (pHH3, mitosis marker) after the tumor volume had been reduced to approximately half of its initial size. Compared to the saline group, results indicated that the Sgc8c-M-treated tumors exhibited a decrease in CK19 expression, a reduction in Ki67 levels, and an increase in pHH3 staining (Fig. 2e, g). These findings imply that tumors after Sgc8c-M treatment showed a remarkable decrease in epithelial cancer cells and cell proliferation, along with an increase in mitotic inhibition, consistent with the effect of tubulin inhibitor MMAE. As shown in Supplementary Fig. 7, the body weights across all treatment groups remained stable throughout the dosing and subsequent observation periods.

### Strong antitumor effect in pancreatic cancer models

**Fig. 3:**
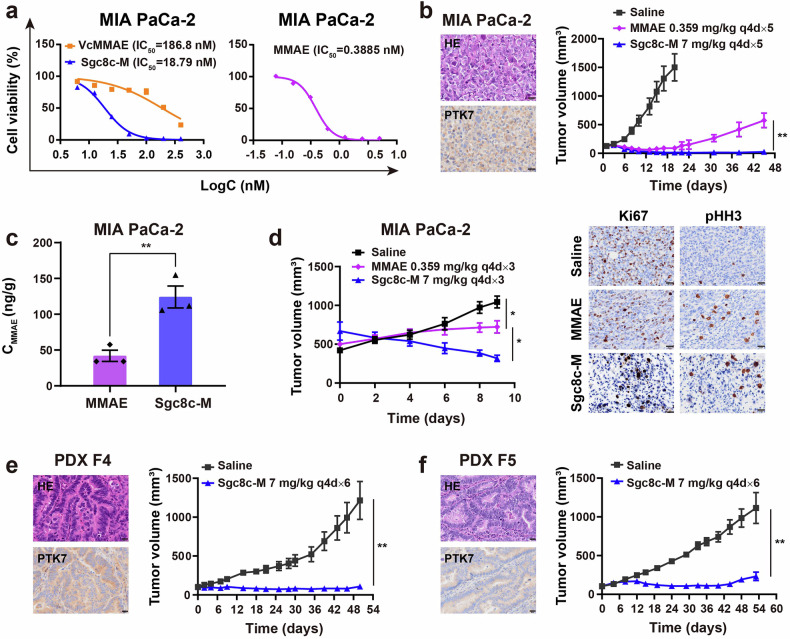
Antitumor effect of Sgc8c-M in pancreatic cancer. **a** Cytotoxicity of VcMMAE, Sgc8c-M, and MMAE in MIA PaCa-2 cells after a 72-h incubation (*n* = 3). **b**, **e**, **f** Antitumor effects of Sgc8c-M for MIA PaCa-2, PDX F4, and PDX F5 in vivo (*n* = 5, unpaired t test). Tumor-bearing mice were dosed by i.v. every four days for 5–6 cycles with 7 mg/kg Sgc8c-M or 0.359 mg/kg MMAE. PTK7 IHC characterization and hematoxylin-eosin (HE) staining on these tumors showed the differences in PTK7 expression and tissue structure. Scale bars, 20 μm. **c** MMAE concentration in MIA PaCa-2 tumors at 2 h after a single dose of 7 mg/kg Sgc8c-M or 0.359 mg/kg MMAE (*n* = 3, unpaired t test). **d** Tumor-killing effect study of Sgc8c-M and MMAE in MIA PaCa-2 xenografts. Mice received 7 mg/kg Sgc8c-M or 0.359 mg/kg MMAE every four days for three cycles (q4d × 3) with Ki67 and pHH3 analyzed 24 h after the final dose (*n* = 3, one-way ANOVA). Scale bars, 20 μm. Data are presented as mean ± SEM. *P < 0.05, **P < 0.01

Pancreatic cancer is currently one of the most challenging malignant tumors, necessitating the urgent development of more effective therapeutic options. PTK7 is reported to be overexpressed in pancreatic cancer, particularly in metastatic tumors, which exhibit a higher positivity rate (60%) compared to primary tumors (40%).^22^ Additionally, PTK7 expression is found in the stromal cells of pancreatic tumors,^17,22^ making it an attractive target for drug delivery in pancreatic cancer therapies. In our investigation of Sgc8c-M for pancreatic cancer therapy, we first evaluated its binding to the MIA PaCa-2 cell line. Cy5-labeled Sgc8c-M and Sgc8c demonstrated high specificity in binding to MIA PaCa-2 cells compared to controls Ctrl-M and Ctrl (Supplementary Fig. 8a). Similar to observations in TNBC cells, Sgc8c-M exhibited a greater cytotoxicity than that of unconjugated VcMMAE. In vitro cytotoxicity revealed that the IC_50_ value of MMAE against MIA PaCa-2 cells was approximately 48-fold lower than that of Sgc8c-M, which could be attributed to MMAE’s ability to diffuse freely into tumor cells without the need for linker cleavage (Fig. 3a). Interestingly, in vivo studies demonstrated that Sgc8c-M achieved superior tumor inhibition in MIA PaCa-2 xenografts compared to the MMAE group at an equivalent dose (Fig. 3b). Quantification of MMAE in tumors showed that Sgc8c-M selectively delivered more MMAE to MIA PaCa-2 tumors at 2 h compared to the MMAE group (Fig. 3c), elucidating the observed difference in in vivo efficacy. Further investigation into the tumor-killing effect of Sgc8c-M in this CDX model revealed decreased Ki67 levels and increased pHH3 staining in tumor sections, suggesting that Sgc8c-M exerts antitumor activity by inducing cell death through mitotic inhibition similar to the mitotic inhibition seen in free MMAE (Fig. 3d). We then assessed the efficacy of Sgc8c-M in two additional PDX models, PDX F4 and PDX F5, and in both models, it demonstrated impressive tumor inhibition with TGI values of 91% and 79%, respectively (Fig. 3e, f). Body weight changes in the aforementioned pancreatic cancer models are presented in Supplementary Fig. 8b–d, showing that no weight loss occurred in any group throughout the treatment and subsequent observation periods.

### Remarkable antitumor effect in other cancer models

In addition to the two cancer types studied above, PTK7 overexpression has been identified in a variety of other malignancies, including CRC, NSCLC, and OVCA.^17,20,29^ Consequently, we also investigated the antitumor efficacy of Sgc8c-M in these specific cancer types.

**Fig. 4:**
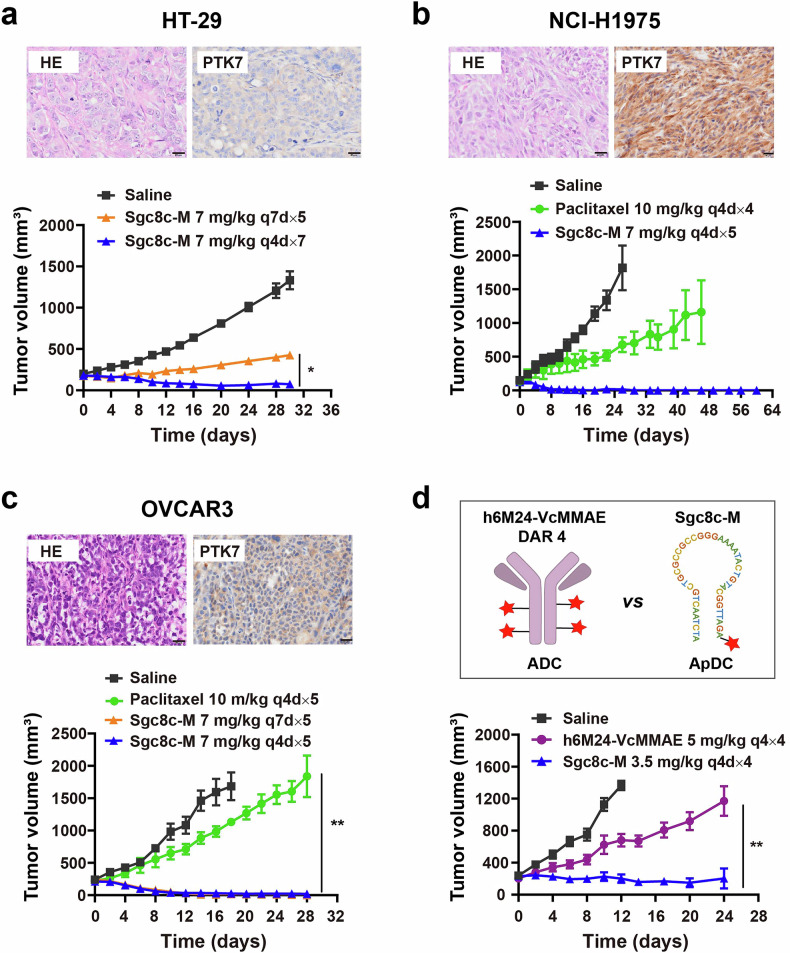
Antitumor activity of Sgc8c-M in HT-29, NCI-H1975, and OVCAR3 xenograft models. In vivo therapeutic efficacy of Sgc8c-M in HT-29 (*n* = 4, one-way ANOVA) (**a**), NCI-H1975 (*n* = 3) (**b**), and OVCAR3 (*n* = 3, one-way ANOVA) (**c**) xenografts. Tumor-bearing mice were dosed with 7 mg/kg Sgc8c-M (i.v.) or 10 mg/kg paclitaxel (i.p.) when the tumor size reached an average of 100–200 mm^3^. Histology and PTK7 expression in these models were shown as HE staining and IHC staining on tumor tissues of untreated mice, respectively. **d** The antitumor effects of Sgc8c-M (3.5 mg/kg) and PTK7-targeted ADC h6M24-VcMMAE (DAR4, 5 mg/kg) in the OVCAR3 model (*n* = 5, unpaired t test). Scale bars, 20 μm. Data are presented as mean ± SEM. *P < 0.05, **P < 0.01

In the PTK7-overexpressing HT-29 CRC model, we initially evaluated the specific recognition ability of Sgc8c-M to the cells through flow cytometry assays. The results demonstrated that both Cy5-labeled Sgc8c and Sgc8c-M exhibited significantly higher fluorescence intensities compared to controls Cy5-Ctrl and Cy5-Ctrl-M in HT-29 cells (Supplementary Fig. 9a). To further investigate the impact of aptamer conjugation on cytotoxicity, we compared the 72-h cytotoxicity of VcMMAE (without aptamer) and Sgc8c-M. As illustrated in Supplementary Fig. 9d, Sgc8c-M displayed approximately 9.14 times greater toxicity towards HT-29 cells than VcMMAE with IC_50_ values of 22.23 nM for Sgc8c-M versus 203.2 nM for VcMMAE. This enhanced cytotoxicity is likely a result of increased recognition and internalization of the conjugate owing to the presence of aptamer Sgc8c, facilitating the rapid release of free MMAE within lysosome to exert cytotoxicity. In vivo assessments revealed that treatment with Sgc8c-M at a dosage of 7 mg/kg every seven days for five cycles (q7d × 5) resulted in a significant 68% reduction in tumor size compared to the saline control group after 28 days of treatment. Notably, administration of Sgc8c-M at 7 mg/kg every four days for seven cycles (q4d × 7) nearly induced complete regression of HT-29 tumors, achieving a TGI of 94% (Fig. 4a), further demonstrating the satisfactory efficacy of Sgc8c-M in the CRC model.

In NSCLC, we evaluated the antitumor activity of Sgc8c-M using the PTK7-overexpressing NCI-H1975 xenograft model. In vitro analyses yielded results consistent with those observed in HT-29 cells. Specifically, Cy5-labeled Sgc8c-M was shown to bind to NCI-H1975 cells with high specificity and selectivity, akin to that seen with Sgc8c (Supplementary Fig. 9b). Furthermore, Sgc8c-M demonstrated more potent cytotoxicity against NCI-H1975 cells compared to that of VcMMAE in the 72-h cytotoxicity assays (Supplementary Fig. 9e). In vivo, treatment with Sgc8c-M at a dosage of 7 mg/kg (q4d×5) resulted in complete tumor regression with no tumor recurrence observed 40 days following the cessation of drug administration (Fig. 4b). To further determine the correlation between efficacy and target expression, we evaluated the antitumor activity of Sgc8c-M in PTK7-negative A549 NSCLC models (Supplementary Fig. 10).^22^ Binding assays confirmed minimal binding of Cy5-Sgc8c-M to A549 cells compared to the Cy5-Ctrl-M control (Supplementary Fig. 10b). In vitro, the 72-h cytotoxicity assays revealed about 16-fold reduced potency against A549 cells (IC_50_ = 331.2 nM) versus PTK7-positive NCI-H1975 cells (IC_50_ = 20.45 nM) (Supplementary Fig. 4 and Supplementary Fig. 9e). In vivo, 7 mg/kg Sgc8c-M (q4d × 5) showed modest A549 tumor growth inhibition (Supplementary Fig. 10c). However, it was unable to induce sustained tumor regression, as was observed in the PTK7-positive NCI-H1975 model. Together, compared to the PTK7-negative A549 model, Sgc8c-M showed stronger tumor suppression in the PTK7-overexpressing NCI-H1975 model, both in vitro and in vivo.

For OVCA, we employed the OVCAR3 CDX model characterized by its overexpression of PTK7 and frequent utilization for assessing the therapeutic efficacy of PTK7-targeted ADCs, including h6M24-vc0101 and MTX-13 (Supplementary Fig. 9c).^17,22^ Results of the 72-h cytotoxicity assay mirrored those observed in HT-29 and NCI-H1975 cells with IC_50_ values of 27.94 nM for Sgc8c-M versus 105.3 nM for VcMMAE (Supplementary Fig. 9f). Treatment with Sgc8c-M at both 7 mg/kg every four days for five cycles (q4d × 5) and 7 mg/kg every seven days for five cycles (q7d × 5) led to a sustained regression of in vivo OVCAR3 tumors when compared to that realized with paclitaxel (Fig. 4c). These findings suggest that Sgc8c-M exhibits greater therapeutic efficacy than h6M24-vc0101 (3 mg/kg, q4d × 4) in the treatment of OVCAR3 tumors in which rapid tumor recurrence was observed in the literature.^17^ This result encouraged the construction of a class of ADC h6M24-VcMMAE using the antibody in h6M24-vc0101 conjugated with the same MMAE payload in Sgc8c-M for further comparison. The *K*_d_ value of h6M24-VcMMAE to the human PTK7 protein was determined to be 3.18 nM (Supplementary Fig. 11a), which is comparable to the binding affinity of h6M24-vc0101 in literature. Impressively, as demonstrated in Fig. 4d, the treatment by Sgc8c-M (3.5 mg/kg, q4d × 4) achieved significant stronger tumor inhibition compared to that of h6M24-VcMMAE (5 mg/kg, q4d × 4) in OVCAR3 model.

Collectively, these findings illustrate the substantial therapeutic potential of Sgc8c-M in addressing CRC, NSCLC, and OVCA. Sgc8c-M exhibited potent antitumor effects across these models, with mice body weights remaining stable throughout the treatment and subsequent observation periods (Supplementary Fig. 11b and Supplementary Fig. 12a-c). Following the study of the antitumor efficacy of Sgc8c-M, we conducted further investigations into its tumor-killing effects through IHC characterization of Ki67 and pHH3. Treatment with Sgc8c-M commenced when tumor volumes reached approximately 500 mm³. After administering 3 to 4 doses of our ApDC, tumor volumes exhibited an average reduction of approximately 35–50% (Supplementary Fig. 12d–f). In the evaluated models of HT-29, NCI-H1975, and OVCAR3, IHC characterization revealed that tumor tissues subjected to Sgc8c-M treatment exhibited a significant decrease in Ki67 expression and an increase in pHH3 staining. These results suggest that Sgc8c-M effectively decreases tumor cell proliferation and promotes an increase in mitotic arrest.

To investigate the reasons for the greater efficacy of Sgc8c-M than h6M24-VcMMAE, we conducted a comparative analysis of tumor MMAE levels in OVCAR3 tumor-bearing mice following a single administration of the two drugs. Quantitative analysis revealed significantly higher MMAE levels (normalized as percent of injected dose per gram, %ID/g) in tumors at both 2 h and 6 h post-administration for Sgc8c-M compared to h6M24-VcMMAE (Supplementary Fig. 13). These findings demonstrate that Sgc8c-M achieves more rapid and higher tumor accumulation than h6M24-VcMMAE, thus producing better efficacy.

### Pharmacokinetic profiles of Sgc8c-M in mice, rats, and cynomolgus monkeys

**Fig. 5:**
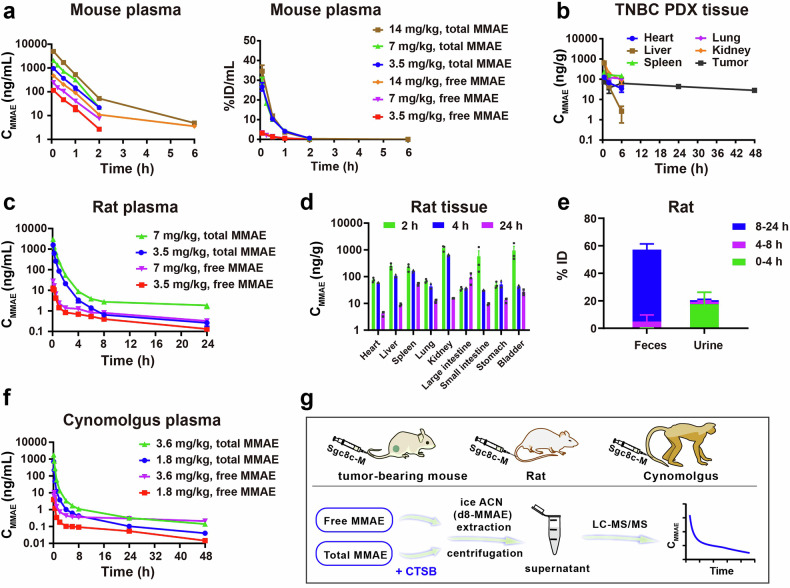
Pharmacokinetic (PK) study of Sgc8c-M in mice, rats, and cynomolgus monkeys. **a** PK profiles and %ID/mL of free and total MMAE in plasma of mice after intravenous administration of 3.5, 7, and 14 mg/kg Sgc8c-M. **b** Total MMAE in major tissues of TNBC PDX-bearing mice after intravenous administration of 7 mg/kg Sgc8c-M. **c** Free and total MMAE in plasma of rats after intravenous injection of 3.5 and 7 mg/kg Sgc8c-M. **d** Total MMAE in tissues of rats at 2 h, 4 h, and 24 h after intravenous injection of 7 mg/kg Sgc8c-M. **e** Excretion of total MMAE in rats within 24 h after intravenous injection of 7 mg/kg Sgc8c-M. **f** Free and total MMAE in plasma of cynomolgus monkeys after intravenous injection of 1.8 and 3.6 mg/kg Sgc8c-M. **g** Schematic diagram illustrating the determination of free and total MMAE levels. Data are presented as mean ± SEM (*n* = 3)

Building upon the promising therapeutic efficacy of Sgc8c-M across multiple tumor models, we further investigated the pharmacokinetics (PK) and toxicokinetics (TK) profiles of Sgc8c-M, noting that previously reported ApDCs largely ignored these assays. In the present study, we analyzed PK and TK results to understand the absorption, distribution, metabolism, and elimination of Sgc8c-M. Such studies would ultimately aid in the optimization of therapeutic regimens and assessment of safety profile for potential clinical application.^30,31^ Here, we first conducted a systematic evaluation of the PK profiles by quantifying both free and total MMAE in plasma, as well as total MMAE levels in various tissues, employing liquid chromatography-tandem mass spectrometry (LC-MS/MS) across different species, including mice, rats, and cynomolgus monkeys. To effectively measure free MMAE, we utilized an extraction protocol using ice acetonitrile (ACN) to directly extract cleaved MMAE to the organic phase. In contrast, the measurement of conjugated MMAE required an additional linker-cleaving step using CTSB or liver homogenate. Therefore, total MMAE (conjugated MMAE + free MMAE) could be measured by first cleaving the linker, followed by extraction with ACN (Fig. 5g).

In mice, a notable dose correlation of free and total MMAE was observed at three dosage levels (3.5, 7, and 14 mg/kg) in plasma such that the percent of injected dose per milliliter (%ID/mL) values displayed close concordance (Fig. 5a). Of particular interest, while initial MMAE concentrations in tumors were relatively low compared to those in normal organs, MMAE levels in tumors remained stable over a 48-h period (Fig. 5b). In contrast, MMAE levels in normal organs were below the limit of quantification (LOQ) by 24 h post-administration. Overall, the PK profiles suggest that Sgc8c-M results in transient MMAE levels in plasma, while sustaining plateau-like MMAE levels in tumors (Supplementary Fig. 14a).

In rats, the PK profiles for doses of 3.5 and 7 mg/kg Sgc8c-M were also dose-dependent with half-lives (t_1/2_) of 8.84 ± 4.78 h and 11.65 ± 8.02 h for total MMAE in plasma, respectively (Fig. 5c, Supplementary Fig. 14b, and Supplementary Table 1). The ratio of the area under the concentration-time curves (AUC) for free MMAE to total MMAE was found to be less than 3% (AUC _free MMAE_/AUC _total MMAE_ < 3%), indicating that Sgc8c-M maintains stability in rat plasma with minimal MMAE leakage (Supplementary Table 1). Comparative analysis revealed that the kidney, spleen, and bladder were the top three tissues with the highest MMAE exposure among rat tissues within 24 h (Supplementary Table 2). Except for the large intestine, MMAE levels in other rat tissues were rapidly metabolized and cleared after 24 h, paralleling observations in mouse models (Fig. 5d). Subsequent investigations into the excretion profile of Sgc8c-M in rats involved quantifying MMAE levels in urine and feces. Results indicated that MMAE was predominantly excreted via urine during the initial 0–4 h, while fecal excretion was more significant between 8 and 24 h (Fig. 5e). Cumulatively, approximately 20.05% of MMAE was excreted in the urine, and around 54.66% was eliminated through feces within 24 h. These findings indicate biliary excretion occurred, thus demonstrating that hepatic metabolism plays an important role in the clearance of Sgc8c-M. Further studies are needed in the future to investigate species-dependent differences in MMAE metabolism.

In cynomolgus monkeys, a similar linear correlation of MMAE PK was observed for both 1.8 mg/kg and 3.6 mg/kg dosages of Sgc8c-M with comparable t_1/2_ for total MMAE of 11.34 ± 0.93 h and 12.85 ± 1.77 h, respectively (Fig. 5f, Supplementary Fig. 14c, and Supplementary Table 1). The AUC _free MMAE_ /AUC _total MMAE_ ratios for the 1.8 mg/kg and 3.6 mg/kg Sgc8c-M groups were measured at 2.1% and 3.1%, respectively (Supplementary Table 1). The low exposure levels of free MMAE in the plasma of cynomolgus monkeys further indicate the high linker stability of Sgc8c-M within this primate model.

### Toxicokinetic and toxicity study of Sgc8c-M in rats and cynomolgus monkeys

**Fig. 6:**
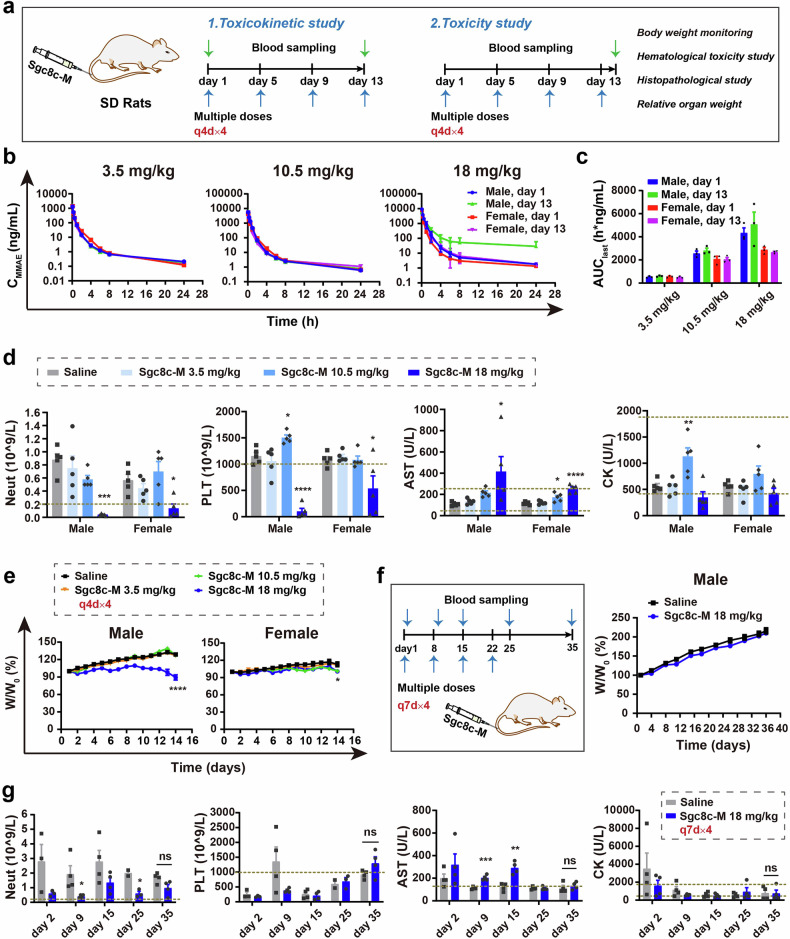
Toxicokinetic (TK) and toxicity study of Sgc8c-M in rats in repeated-dose studies. **a** Schematic illustrates the dosing regimen and blood sampling times in the repeated-dose TK and toxicity study in Sprague-Dawley (SD) rats. **b** Plasma concentration-time curves of total MMAE in rats following intravenous injection of Sgc8c-M at doses of 3.5, 10.5, and 18 mg/kg on days 1 and 13 in the repeated-dose TK study (*n* = 3). **c** AUC values derived from the plasma concentration-time curves presented in Fig. 6b (*n* = 3). **d** Neut, PLT, AST, and CK levels on day 14 (24 h post-administration) in the repeated-dose toxicity study of Sgc8c-M (*n* = 5, one-way ANOVA). **e** W/W_0_ (%) of male and female rats over the course of the repeated-dose toxicity study (*n* = 5, one-way ANOVA). W/W_0_ (%) represented the body weight curve normalized to the initial body weight, where W is the measured weight and W_0_ is the initial body weight. **f** W/W_0_ (%) of male rats after the dosing interval for 18 mg/kg Sgc8c-M was extended from every 4 days (q4d × 4) to every 7 days (q7d × 4). The dosing regimen and blood sampling times are indicated on the left side (*n* = 4). **g** Neut, PLT, AST, and CK levels in male rats after the extended dosing interval of 18 mg/kg Sgc8c-M (*n* = 4, unpaired t test). Data are presented as mean ± SEM. *P < 0.05, **P < 0.01, ***P < 0.001, ****P < 0.0001, ns not significant

**Fig. 7:**
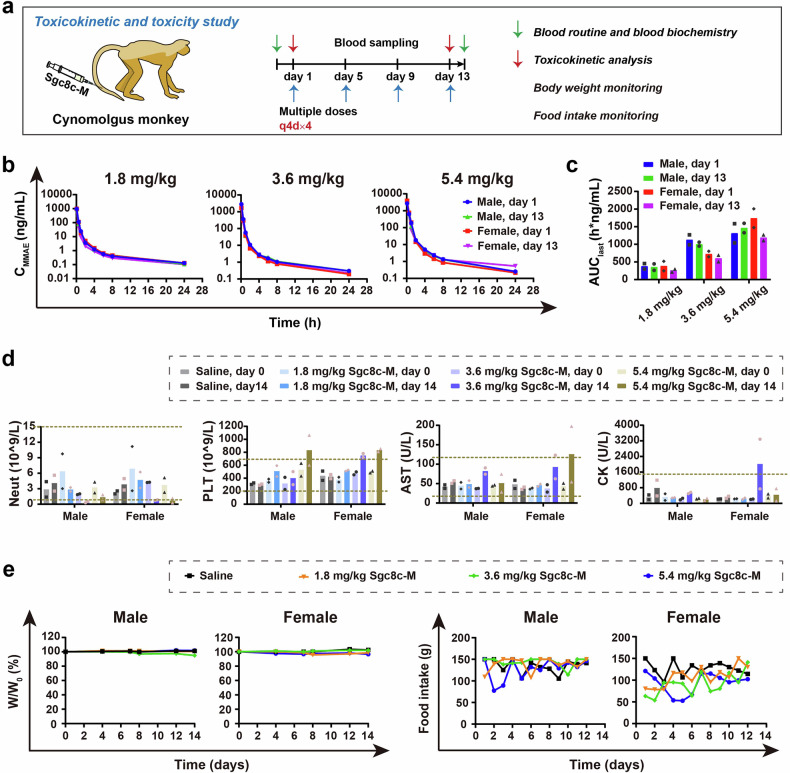
Toxicokinetic (TK) and toxicity study of Sgc8c-M in cynomolgus monkeys. **a** Schematic represents the dosing regimen and blood sampling time in the repeated-dose TK and toxicity study in cynomolgus monkeys. **b** Plasma concentration-time curves of total MMAE in cynomolgus monkeys after intravenous injection of Sgc8c-M at doses of 1.8, 3.6, and 5.4 mg/kg on days 1 and 13 in the repeated-dose TK study. **c** AUC values obtained from the plasma concentration-time curves shown in Fig. 7b. **d** Blood routine and blood biochemistry parameters on days 0 (24 h before the repeated dose) and 14 (24 h after the repeated dose) in the repeated-dose toxicity study. **e** W/W_0_% (left) and food intake (right) of cynomolgus monkeys after intravenous injection of Sgc8c-M at three doses (1.8, 3.6, and 5.4 mg/kg) in the repeated-dose toxicity study. Data are presented as the mean of two cynomolgus monkeys

Subsequent investigations were focused on the toxicokinetics and toxicity of Sgc8c-M in rats and cynomolgus monkeys in repeat-dose studies (once every 4 days for four cycles, q4d × 4) with doses up to 18 mg/kg in rats and 5.4 mg/kg in monkeys (Figs. 6, 7). Total MMAE toxicokinetic profiles for rats and cynomolgus monkeys are depicted in Figs. 6b, 7b, respectively. Major toxicokinetic parameters were calculated from a non-compartmental analysis (PK solver 2.0, an add-in program in Microsoft Excel) of the samples obtained following intravenous bolus input. Comprehensive details of the TK data are outlined in Supplementary Tables 3 and 4.

Following the administration of Sgc8c-M to rats at doses of 3.5, 10.5, and 18 mg/kg, both single-dose (day 1) and multiple-dose (day 13, the fourth cycle) resulted in systemic MMAE exposure, which exhibited a dose-dependent increase alongside a linear toxicokinetic profile (Fig. 6b, c and Supplementary Fig. 15a, b). Notably, the exposure levels of MMAE were comparable following the first and fourth doses, indicating that no significant drug accumulation occurred after multiple administrations (Fig. 6c and Supplementary Fig. 15c).

In the repeated-dose toxicity study, the hematological changes of Sgc8c-M were dose-dependent, and hematotoxicity manifested at the highest dosage of 18 mg/kg in rats. As illustrated in Fig. 6d, significant reductions in neutrophil (Neut) and platelet (PLT) levels were observed in the 18 mg/kg Sgc8c-M group. No significant alterations in hematological parameters were evident in the 3.5 mg/kg Sgc8c-M group relative to the corresponding saline controls of the corresponding gender, suggesting that the dose shown to be efficacious in mouse models (7 mg/kg in mice, equivalent to 3.5 mg/kg in rats) does not induce hematotoxicity in rats (Fig. 6d and Supplementary Fig. 16). Additionally, alterations in white blood cell (WBC) subsets (including Neut, lymphocyte (Lymph), monocyte (Mono), and eosinophil (Eos)) and red blood cell (RBC) subsets (including hemoglobin (HGB), hematocrit (HCT), and reticulocyte (Retic)) were observed in both the 10.5 mg/kg and 18 mg/kg Sgc8c-M groups, demonstrating a dose-dependent decrease consistent with changes in overall WBC and RBC (Supplementary Fig. 16). As illustrated in Fig. 6d and Supplementary Fig. 17, both male and female rats treated with 3.5 mg/kg Sgc8c-M maintained normal liver function, with no significant differences in major liver function biomarkers verse saline controls of the same gender. At 10.5 mg/kg, male rats showed significant decrease in total protein (TP), while female rats exhibited elevated serum aspartate transaminase (AST). Following administration of 18 mg/kg Sgc8c-M, male rats demonstrated significant elevations in AST, alanine aminotransferase (ALT), total bilirubin (TBIL), and globulin (GLB) levels, alongside reductions in albumin (ALB) and ALB/GLB (A/G). In contrast, female rats showed significant increases in AST, ALT, and gamma-glutamyl transferase (GGT). These findings indicate that repeated administration of 3.5 mg/kg Sgc8c-M (equivalent to the highly efficacious therapeutic dose of 7 mg/kg in mice) did not affect liver function. Effects on liver function were observed only at the higher doses of 10.5 mg/kg and 18 mg/kg, aligning with MMAE’s established hepatotoxicity profile. This conclusion was further corroborated by significant increases in relative liver weight in the 10.5 mg/kg and 18 mg/kg groups (Supplementary Fig. 18). Histopathological examination of hematoxylin and eosin (HE)-stained liver sections from male rats treated with 18 mg/kg Sgc8c-M revealed mild hepatotoxicity characterized by slight hepatocyte necrosis and inflammatory cell infiltration (Supplementary Fig. 19). Furthermore, HE staining of heart tissue from male rats in this treatment group revealed partial atrophy of myocardial fibers accompanied by widened gap and mild inflammation.

In the context of body weight monitoring, male rats treated with 18 mg/kg Sgc8c-M exhibited a weight loss exceeding 15% (n = 2/5), while female rats administered 10.5 mg/kg and 18 mg/kg Sgc8c-M showed only slight weight loss (Fig. 6e). To further investigate the toxicity in male rats, we subsequently modified the dosing schedule for male rats receiving 18 mg/kg Sgc8c-M from a regimen of once every four days for four cycles (q4d × 4) to once every seven days for the same number of cycles (q7d × 4). Notably, this adjustment resulted in no significant reduction in body weight, as well as the absence of severe hematological and hepatic toxicity, since blood count and chemistry parameters returned to normal during the recovery period (Fig. 6f, g and Supplementary Fig. 20), suggesting that the toxicity caused by the administration of 18 mg/kg Sgc8c-M in male rats is reversible.

When synthesizing the findings from routine blood tests, blood biochemical markers, relative organ weights, histopathological examinations, and body weight assessments in the repeated-dose toxicity study, we can conclude that a dose of 3.5 mg/kg Sgc8c-M (equivalent to 7 mg/kg in mouse models, which has been shown to be highly efficacious) is both safe and effective without eliciting toxicity in rats. At the 10.5 mg/kg dosage, minor hematological and hepatic toxicities were observed; however, this dosage was generally well tolerated by rats. Conversely, the 18 mg/kg dosage of Sgc8c-M presented a higher toxicity profile, especially in male rats, while females tolerated this dose well. These observations underscore the necessity of considering gender differences in toxicological responses, particularly at elevated dosage levels. Our findings revealed that the toxicity associated with Sgc8c-M is consistent with the mechanism of pharmacological activity of MMAE. Furthermore, ApDC does not present any obvious novel mechanism.

The exposure levels of MMAE in cynomolgus monkeys demonstrated a dose-dependent increase following the administration of Sgc8c-M across a range of dosages from 1.8 mg/kg to 5.4 mg/kg with similar %ID/mL-time curves (Fig. 7b, c and Supplementary Fig. 21a, b). Consistent with the findings in rats, MMAE exposure in cynomolgus monkeys after the first dose was comparable to that observed following the fourth dose, suggesting that no significant drug accumulation occurred at any administered dosage (Fig. 7c and Supplementary Fig. 21c).

In the repeated-dose toxicity study involving cynomolgus monkeys, most parameters from routine blood tests and biochemical blood analyses remained well within the normal reference ranges (Fig. 7d, Supplementary Fig. 22, and Supplementary Fig. 23). Our analysis specifically focused on variations in PLT and Neut levels. Across the 1.8–5.4 mg/kg dosing regimen, PLT levels showed no reduction following repeated Sgc8c-M administration compared to pre-administration (Fig. 7d). Conversely, decreased Neut levels were observed, consistent with the known hematological toxicity of MMAE. The liver and kidney functions of cynomolgus monkeys were maintained within normal limits. With respect to body weight and food intake, the body weights of the cynomolgus monkeys remained stable across all three dosages. Although fluctuations in food intake were noted during the administration period, consumption levels normalized at the end of administration (Fig. 7e). Collectively, these findings indicate that Sgc8c-M exhibits a favorable safety profile within the examined dose range of 1.8 to 5.4 mg/kg in cynomolgus monkeys.

### Maximum tolerated dose study

The biggest limitation to clinical application of MMAE-based therapeutic agents is their pronounced toxic effects.^32–34^ Thus, we conducted a maximum tolerated dose (MTD) study of a single injection of Sgc8c-M in mouse, rat, and cynomolgus monkey models. The findings, as depicted in Supplementary Fig. 24, indicate that the MTD of Sgc8c-M ranged from 24.4 mg/kg to 28.1 mg/kg in female mice. In rat models, the MTD for male and female subjects was determined to be 27.5–30 mg/kg and 24–27.5 mg/kg, respectively. For cynomolgus monkeys, the MTD of Sgc8c-M exceeded 12.5 mg/kg for both male and female monkeys. It has been demonstrated that rodent-specific carboxylesterase 1c (CES 1c) influences the stability of the valine-citrulline-p-aminobenzyloxycarbonyl (VC-PAB) linker and that this effect is more pronounced in mice.^35,36^ The elevated MTDs of Sgc8c-M observed in rats and cynomolgus monkeys compared to mice can be attributed to the enhanced stability of the VC linker in these species. Previous studies reported that a single administration of 0.16 mg/kg MMAE resulted in 100% mortality in cynomolgus monkeys, while a single injection of 18 mg/kg Disitamab vedotin (equivalent to approximately 0.35 mg/kg MMAE) led to 50% mortality.^37^ Encouragingly, in our investigations, cynomolgus monkeys treated with 12.5 mg/kg of Sgc8c-M, which corresponds to approximately 0.63 mg/kg MMAE, exhibited no observable abnormalities. These comparative analyses suggest a significant improvement in acute tolerance to MMAE when conjugated to aptamer Sgc8c, implying that the ApDC demonstrates enhanced safety in cynomolgus monkeys compared to the clinically approved ADC, Disitamab vedotin.

Furthermore, the minimum effective dose (MED) of Sgc8c-M in mouse models was established at 3.5 mg/kg to 7 mg/kg. This indicates that our ApDC possesses a wider therapeutic window [therapeutic window = (MTD of Sgc8c-M in cynomolgus monkeys)* 4/ (MED of Sgc8c-M in mice), calculated as 12.5*4/7 to 12.5*4/3.5, resulting in a range of 7 to 14] compared to the PTK7-targeted ADC h6M24-vc0101, which has a therapeutic window of approximately 3 to 5.^22^ The broader therapeutic window associated with Sgc8c-M underscores its promising potential for clinical translation, as well as the capacity for expanding the population of patients who may benefit from this therapeutic approach.

## Discussion

Sgc8c-M represents a PTK7-targeted ApDC that exhibits significant antitumor efficacy, favorable pharmacokinetic and toxicokinetic profiles, as well as a commendable safety and extended therapeutic window in cynomolgus monkeys, a relevant non-human primate model. Sgc8c-M is composed of a high-affinity, PTK7-specific aptamer, Sgc8c, a dipeptide VC-based linker, and the microtubule inhibitor MMAE. PTK7 is overexpressed in various cancers and is associated with oncogenic functions;^21,38^ however, the development of blocking antibodies or small-molecule inhibitors has been challenging owing to the lack of catalytic activity of PTK7. The ApDC Sgc8c-M could effectively circumvent these limitations, achieving substantial tumor inhibition.

The production of Sgc8c-M can be performed as a homogeneous drug with high yield and purity via a one-step Michael addition reaction, followed by efficient separation and purification through HPLC. This straightforward preparation and purification process is advantageous for scaling up and facilitating industrial manufacturing of ApDCs. It is worth mentioning that Sgc8c-M can recognize PTK7 proteins across various species, including mice, rats, cynomolgus monkeys, and humans, providing a unique advantage over ADCs, some of which can only recognize human proteins. The cross-species recognition of Sgc8c is attributable to the high sequence similarity of the Ig3 to Ig4 binding region on the PTK7 proteins of various species.^39^ Analysis of sequence similarity between human PTK7 and its mouse, rat, and cynomolgus monkey orthologs (sequences obtained from UniProt) in this binding region revealed similarity values of 89.07%, 89.62%, and 96.72%, respectively. This high conservation could enable Sgc8c-M to recognize PTK7 across these species. This cross-species recognition suggests that the potency of Sgc8c-M is likely to remain consistent, enhancing the relevance of our preclinical evaluations. In vivo, Sgc8c-M has displayed high potency, surpassing standard-of-care chemotherapy agents such as paclitaxel. Significant tumor growth inhibition and even tumor regression were observed with an optimized dosing procedure. Additionally, certain sensitive models demonstrated robust responses to lower doses, such as 3.5 mg/kg administered every four days for four cycles (q4d × 4) or 7 mg/kg administered every seven days for three to five cycles (q7d × 3-5). In the OVCAR3 model, Sgc8c-M outperforms the PTK7-targeted ADC h6M24-VcMMAE with the same MMAE payload, further validated the therapeutic potential of ApDC as an emerging targeted antitumor agent. Preliminary analyses indicate that the efficacy of Sgc8c-M appears to depend on PTK7 expression levels in different tumor models of the same cancer type, including TNBC, pancreatic cancer, and NSCLC (Supplementary Table 7 and Supplementary Fig. 25). However, efficacy may also be influenced by other factors such as cancer type, cancer cell sensitivity, and tumor heterogeneity.

Besides its high potency, Sgc8c-M was well tolerated and demonstrated a favorable safety profile in cynomolgus monkeys with an expanded therapeutic window ranging from 7 to 14 when compared to the therapeutic window of 3 to 5 observed for the PTK7 ADC h6M24-vc0101.^22^ This enhanced safety of Sgc8c-M may be attributed to the PK profile of rapid clearance in normal tissues and the TK profile of no drug accumulation after multiple administrations. In tumor-bearing mice, Sgc8c-M enabled prolonged delivery of MMAE to the tumor site following a transient exposure in plasma, exhibiting a plateau-like pharmacokinetic profile with MMAE concentrations persisting beyond 48 h in tumors. Furthermore, Sgc8c-M was rapidly cleared from normal tissues in mice and rats within a 24-h window. These PK profiles contributed to effective tumor-killing, while minimizing toxic side effects. As reported, normal tissue expression does not consistently predict targeted drug toxicity, particularly for microtubule inhibitor payloads, which require high antigen expression and actively cycling cells to be effective.^17,40^ Collectively, these properties result in minimal target-dependent toxicity in normal tissues with low PTK7 expression. The acute tolerance to MMAE was significantly enhanced by its conjugation to the aptamer Sgc8c. Notably, no significant abnormalities were observed in cynomolgus monkeys administered with 12.5 mg/kg of Sgc8c-M (containing 0.63 mg/kg MMAE), in contrast to previous reports indicating that the administration of 0.16 mg/kg MMAE resulted in 100% mortality in cynomolgus monkeys.^37^ In the multiple-dose toxicokinetic study, the AUC values following the first and last administrations of Sgc8c-M were not significantly different, indicating the absence of drug accumulation across dosing cycles and the low incidence of antidrug antibodies (ADA) effect in both rats and monkeys.^31,37^ The multiple-dose toxicity assessment of Sgc8c-M in cynomolgus monkeys, across a dose range of 1.8 to 5.4 mg/kg, revealed essentially no adverse effects beyond a reduction in neutrophil levels consistent with the reported MMAE toxicity. Additionally, blood biochemical parameters, body weights, and food intake remained within normal ranges throughout the study.

Despite the fact that the present study systematically evaluated the antitumor efficiency, pharmacokinetics, toxicokinetics, and safety of Sgc8c-M from rodents to non-human primates, establishing its potential as a targeted therapeutic candidate, several limitations warrant acknowledgment. Initial studies of the Cy5-Sgc8c-M endocytosis pathway utilized classical chemical inhibitions. Further validation, particularly through siRNA-mediated knockdown or genetic depletion of key endocytosis regulators, will be critical. Systematic PTK7 expression assessment in CDX and PDX models is essential to clarify the impact of tumor heterogeneity on efficacy. Additionally, long-term toxicity studies are necessary to resolve uncertainties regarding prolonged Sgc8c-M effects on hematopoietic and hepatic function. Further in-depth studies are needed to investigate potential resistance mechanisms, such as PTK7 downregulation or drug efflux mediated by multidrug resistance (MDR) transporters (e.g., ATP-binding cassette subfamily G member 2 (ABCG2) and P-glycoprotein (P-gp)), as well as whether Sgc8c-M induces immunogenic cell death and activates associated immune pathways. Ultimately, these investigations will be valuable in promoting the future clinical translation of Sgc8c-M. Bispecific targeting strategies may overcome the efficacy limitations of monospecific agents imposed by tumor heterogeneity and drug resistance, with reduced off-target toxicity.^41^ As exemplified by BCG033, a bispecific ADC targeting both trophoblast cell surface antigen 2 (TROP2) and PTK7, this approach has demonstrated enhanced therapeutic efficacy with reduced off-target effects in multiple tumor models.^42,43^ Notably, the high programmability and ease of engineering of aptamers enable efficient construction of bispecific ApDCs, providing strong rationale for their development in our future research.

In conclusion, we have successfully developed a robust PTK7-targeted ApDC, characterized by broad and potent antitumor effects with good safety profiles. The clinical efficacy of h6M24-vc0101 has demonstrated PTK7 as a promising therapeutic target. Sgc8c-M exhibits superior preclinical efficacy in certain models and possesses a broader therapeutic window compared to h6M24-vc0101. These preclinical results suggest Sgc8c-M may offer a novel treatment option for PTK7-expressing solid tumors in clinical settings. The innovative construction of ApDC together with comprehensive evaluation from rodents to non-human primates in this study holds great promise to advance aptamer-based targeted cancer therapies for clinical translation to benefit patients with improved treatment outcomes.

## Materials and methods

### Cell culture

All cell lines used in this study were purchased from the American Type Culture Collection (ATCC) or cell bank of the Chinese Academy of Sciences (Shanghai, China). The cells were maintained in DMEM (for SUM159, MDA-MB-468, MIA PaCa-2, OVCAR3, and IOSE80) or RPMI-1640 (for Ramos, A549, and NCI-H1975) or DMEM/F12 (for HT-29) medium supplemented with 10% fetal bovine serum and 1% penicillin-streptomycin. All cells were cultured in an incubator at 37 °C, 5% CO_2_.

### Synthesis of Sgc8c-M

Sgc8c-SH was synthesized using modules 3’-thiol modifier C6 S-S controlled pore glass (CPG) and phosphoramidites A, T, C, and G on an automated DNA synthesizer. The molecular assembly of ApDC consisted of Sgc8c-SH in double distilled water (ddH_2_O) added into VcMMAE (MCE) in ACN at a molar ratio of DNA to VcMMAE of 1:10 with a solvent ratio of water to ACN of 1:1. The mixture was shaken at 37 °C for 3 h to facilitate the coupling reaction. The resulting product, Sgc8c-M, was purified using HPLC and characterized via mass spectrometry to confirm its identity and purity.

### Synthesis of h6M24-VcMMAE

The PTK7-targeted ADC h6M24-VcMMAE (drug-antibody ratio, DAR4) was synthesized by AISEE Biotechnology.

### Cell binding assay

Adherent cells were harvested and counted after digestion with non-enzyme cell detach solution, while suspended Ramos cells were directly counted as 2 × 10^5^ cells/ sample. Subsequently, cells were resuspended in a binding buffer (washing buffer supplemented with 0.1 mg/mL of yeast tRNA and 1 mg/mL of bovine serum albumin (BSA)) and incubated with Cy5-labeled Sgc8c, Ctrl, Sgc8c-M, and Ctrl-M (final concentration: 250 nM) at 4 °C for 30 min. After washing three times through centrifugation (4 °C, 1000 rpm, 3 min) using washing buffer (Dulbecco’s phosphate-buffered saline (DPBS) supplemented with 5 mM of MgCl_2_ and 4.5 g/L of glucose), cells were dispersed in 200 μL washing buffer for subsequent flow cytometry assay.

### Binding assay of Sgc8c-M to PTK7 by SPR

For SPR analysis, human (#19399-H08H, Sino Biological), mouse (HY-P78761, MCE), rat (HY-P78619, MCE), and cynomolgus monkey (#90979-C08H, Sino Biological) PTK7 proteins with His tags were immobilized on a carboxymethylated dextran CM5 chip on SPR (Cytiva Biacore 8 K). Sodium acetate buffer (NaAC) at pH 5.0/5.5 was used for human and cynomolgus monkey PTK7 proteins, while a pH of 4.5 was applied for mouse and rat PTK7 proteins. His protein was simultaneously immobilized on the CM5 chip as a control using pH 4.5 NaAC. The final concentration of all above proteins was 10 μg/mL. Unreacted carboxyl groups were subsequently deactivated using ethanolamine-HCl. Sgc8c were diluted to 25, 50, 100, 200, and 400 nM in running buffer (DPBS supplemented with 5 mM MgCl_2_), and Sgc8c-M and Ctrl-M were diluted to 400 nM. Sgc8c, Sgc8c-M, and Ctrl-M were sequentially injected over the immobilized surface from low to high concentrations for a 180-s association phase followed by a 180-s dissociation phase at a flow rate of 10 µL/min. The capture surface was regenerated after each cycle with a 30-s contact time of 1.5 M NaCl at a flow rate of 30 µL/min. The equilibrium dissociation constant (*K*_d_) was subsequently analyzed by Biacore Insight Evaluation software.

### Cell viability

Cells were seeded in wells of a 96-well plate (6000 cells/well) and incubated overnight to allow for cell adherence. On the second day, 100 μL complete medium containing VcMMAE and Sgc8c-M (400, 200, 100, 50, 25, 12.5, and 6.25 nM), as well as MMAE (5, 2.5, 1.25, 0.625, 0.3125, 0.15625, and 0.078125 nM), were added to wells of a 96-well plate for an additional 72-h incubation at 37 °C. At the end of incubation, 100 μL of CellTiter 96^®^ Aqueous One Solution Cell Proliferation Assay (MTS, Promega) solution (prepared by mixing 2 mL MTS with 9 mL of complete medium) were added to each well. The plates were then incubated for an additional 1–2 h to allow for the formation of formazan crystals. The absorbance of each well was measured at 490 nm using a Multi-Mode Microplate Reader (TECAN, SPARK) to assess cell viability. Cell viability was calculated as [OD_490_ (treatment) - OD_490_ (blank)]/[OD_490_ (nontreatment) - OD_490_ (blank)] × 100%.

### Cell internalization study

#### Internalization behavior studied by confocal imaging

SUM159 cells were seeded in 15 mm confocal dishes (1 × 10^5^ cells/dish) and incubated overnight for cell adherence. The following day, SUM159 cells were incubated with Cy5-Sgc8c-M (final concentration: 400 nM) at 37 °C for 0.5 h and 2 h. Hoechst 33342 (final concentration: 0.1 mg/mL) was added during the last 30 min of incubation for cell nucleus staining. At the end of incubation, the medium was removed, and the cells were washed three times with DPBS. Subsequently, fresh medium containing LysoTracker Green DND-26 (final concentration: 100 nM, Invitrogen) was added and incubated with cells at room temperature for 10 min for lysosome staining prior to confocal imaging.

#### Internalization pathway studied by flow cytometry

SUM159 and MDA-MB-468 cells were seeded in wells of a 24-well plate (1 × 10^5^ cells/well) and allowed to adhere overnight. The following day, cells were preincubated with chemical inhibitors [methyl-β-cyclodextrin (M-β-CD, #M102038, Aladdin), caveolae pathway; chlorpromazine hydrochloride (CPZ, #B1480, APExBIO), clathrin pathway; amiloride hydrochloride dihydrate (AMI, #B2268, APExBIO), macropinocytosis pathway] at 37 °C for 0.5 h prior to the addition of Cy5-Sgc8c-M (final concentration: 400 nM). Cells without preincubation of inhibitors were used as controls. After a 2-h incubation, the cells were washed three times with DPBS, digested with trypsin, and then subjected to three additional washes through centrifugation (25 °C, 1000 rpm, 3 min). Finally, the cells were resuspended in 200 μL DPBS for subsequent flow cytometry assay.

### Animal study

Female BALB/c nude mice (4–6 weeks old) were purchased from Zhejiang Animal Center and housed under pathogen-free conditions. The TNBC PDX model was kindly provided by Yang Yu and Xianghou Xia. Pancreatic cancer PDX F4 and F5 models were generously provided by Liwei Wang. The animal studies on mice were approved by the Institutional Animal Care and Use Committee (IACUC) of Hangzhou Institute of Medicine (HIM), Chinese Academy of Sciences (2022R0015 and AP2024-11-0334). The animal studies on rats and cynomolgus monkeys were approved by the IACUC of Suzhou Frontage New Drug Development Co., Ltd. (AN-2023-M010 for rats’ experiments and AN-2023-M012 for cynomolgus monkeys’ experiments).

### Western blot analysis

#### Western blot analysis on cell samples

Cells were lysed in RIPA buffer (APPLYGEN, #C1053) supplemented with protease inhibitor cocktails at 4 °C for 30 min, with vortexing every 10 min. Following centrifugation at 4 °C, 12,000 rpm for 10 min, the supernatants were collected and protein concentrations were determined using a BCA assay. Equal amounts of lysate were separated in 10% SDS-PAGE and subsequently transferred onto a polyvinylidene fluoride (PVDF) membrane. In order to reduce non-specificity, the PVDF membrane was blocked with protein free rapid blocking buffer for 10 min, followed by washing in TBST three times for 10 min. The membrane was then incubated overnight at 4 °C with the primary antibody against PTK7 (1:1000, Abclonal, # A9839), followed by incubation with the HRP-conjugated secondary antibody (1:2000, CST, #7074) at room temperature for 1 h. After incubation with ECL reagent substrate for 3–5 min, protein bands were visualized using an ImageQuant 800 system (Amersham). Grayscale values of images were analyzed by ImageQuant TL software.

#### Western blot analysis on tumor samples

Tumor samples were collected on ice, then washed with ice-cold DPBS and blotted dry, followed by freezing in liquid nitrogen and storage at −80 °C for further analysis. For preparation of protein extracts, tumor samples were homogenized in ice-cold RIPA buffer supplemented with protease inhibitor cocktails. The debris was removed, and the supernatant was obtained by centrifugation at 4 °C, 12,000 rpm for 30 min. Subsequently, western blot analysis was performed as described in the cellular-level protocol.

### Immunofluorescence

#### Immunofluorescence in SUM159 cells

SUM159 cells were seeded in 15 mm confocal dishes (1 × 10^5^ cells/dish) and incubated overnight for cell adherence. Then, cells were incubated with 400 nM Cy5-Sgc8c-M in binding buffer at 4 °C for 30 min. Following three washes with washing buffer, cells were incubated with Alexa Fluor 488-labeled PTK7 antibody (1:100, biotechne, #NBP2-73708AF488) in binding buffer at 4 °C for another 30 min. After a final three washes with washing buffer, complete culture medium was added, and cells were imaged using a confocal microscopy to investigate the colocalization of Cy5-Sgc8c-M and PTK7 proteins.

#### Immunofluorescence in SUM159 tumors

SUM159 tumor-bearing mice were intravenously injected with 7 mg/kg Cy5-Sgc8c-M, followed by tumors collection at 2 h post-injection for embedding in optimal cutting temperature (OCT) compound. Using a freezing microtome, 10-μm-thick tumor frozen sections were cut, equilibrated to room temperature, and then incubated in PBS for 2 min to remove OCT. Tumor frozen sections were sequentially fixed in cold acetone, acetone/chloroform (1:1, v/v), and acetone for 5 min each. Following fixation, sections were rinsed once with ddH_2_O and then washed three times in PBS. Excess liquid around tumors was carefully aspirated, and tumors were circled by a pap pen. Subsequently, sections were blocked using PBST buffer supplemented with 1% BSA for 30 min at room temperature. After blocking, sections were washed three times in PBS. Sections were then incubated overnight at 4 °C with a Cy3-labeled PTK7 antibody. Unbound antibody was removed by 3× washing with PBS. Finally, an antifade mounting medium containing DAPI was added dropwise to the tumor area. A glass coverslip was placed over sections, and samples were imaged using a confocal microscopy to assess the colocalization of Cy5-Sgc8c-M and PTK7 proteins.

### In vivo fluorescence imaging

Female BALB/c nude mice were subcutaneously (s.c.) injected with MDA-MB-468 cells (1 × 10^7^ cells in a volume of 100 μL, suspended in a mixture of DMEM and Matrigel in 1:1 v/v) into the right armpit of the forelimb. When tumors reached a volume of 300–400 mm^3^, 2 nmol (10 μM, 200 μL) Cy5-Sgc8c-M and Cy5-Ctrl-M were administered into the tumor-bearing mice via tail vein injection. In vivo optical imaging was perfumed using an IVIS Lumina III imaging system at different times (0, 10, 30, 60, 90, and 120 min) post-injection. Following the 120-min imaging session, mice were euthanized, and tumors along with major organs (heart, liver, spleen, lungs, and kidneys) were excised for ex vivo fluorescence imaging.

### In vivo therapeutic evaluations

In vivo therapeutic evaluations were performed to evaluate the antitumor effects of the PTK7-targeted ApDC Sgc8c-M with comparisons made between the chemotherapeutic drug paclitaxel and the ApDC’s toxic payload MMAE in most cases. Sgc8c has been shown to be a biocompatible and non-toxic nucleic acid aptamer in both in vitro and in vivo studies.^12,44^ Therefore, we did not use Sgc8c alone as a control in this study. All tumor models were established on female BALB/c nude mice. Briefly, a 50 μL suspension of tumor cells (SUM159: 5 × 10^6^, MDA-MB-468: 1 × 10^7^, MIA PaCa-2: 5 × 10^6^, OVCAR3: 5 × 10^6^, HT-29: 5 × 10^6^, NCI-H1975: 5 × 10^6^, A549: 5 × 10^6^) mixed with an equal volume of Matrigel (BD) was injected s.c. into the right hind flank region of mice. For the TNBC PDX and pancreatic cancer PDX F4 and F5 models, 3–5 mm^3^ of tumor fragments were implanted into the right hind flank region of mice. When tumor sizes reached approximately 100 mm^3^–300 mm^3^, the mice were randomly divided into control and treatment groups. Sgc8c-M (i.v.), paclitaxel (i.p.), MMAE (i.v.), or ADC h6M24-VcMMAE (i.v.) were administered to tumor-bearing mice respectively, and tumor volume and body weight were measured every 2 to 4 days following the initiation of treatment. Mice were euthanized if tumor volume exceeded 2000 mm^3^, if body weight reduced by more than 15%, or if they exhibited abnormal clinical signs. Tumor volume was calculated according to the following equation: Tumor volume = (tumor length × tumor width^2^)/2. TGI (%) was calculated as follows: TGI (%) = [1 – (mean of tumor volume in the treatment group)/(mean of tumor volume in the saline group)] × 100.

### Immunohistochemistry

PTK7 IHC staining: Formalin-fixed, paraffin-embedded tumor sections (5 μm) were deparaffinized in xylene and rehydrated in graded alcohols followed by pure water. In order to expose the target protein, heat-induced antigen retrieval was performed using Tris-EDTA buffer (pH 9.0) at 95 °C for 20 min and cooled to room temperature. Endogenous peroxidase was inactivated by incubating the sections with 3% hydrogen peroxide for 10 min. To prevent nonspecific binding of the epitope, the sections were blocked with 10% goat serum for 30 min. The primary antibody, anti-PTK7 rabbit polyclonal antibody (#A9839, abclonal), was diluted to a concentration of 1:250 and applied to the sections for overnight incubation at 4 °C. After washing three times, the sections were incubated with HRP-labeled secondary antibody (#DS9800CN, Bond Polymer Refine Detection) for 30 min. PTK7 expression was visualized by microscope (Olympus, VS200) after 3,3’-diaminobenzidine (DAB) and hematoxylin staining.

Ki67, pHH3, and CK19 IHC staining: The staining procedures for Ki67, pHH3, ang CK19 were roughly the same as those for PTK7 with the exception of the antigen retrieval method for pHH3. The primary antibodies of Ki67, pHH3, and CK19 were respectively anti-Ki67 rabbit mAb (#ab16667, Abcam) of 1:500, anti-pHH3 rabbit antibody (#9701S, CST) of 1:200, and anti-CK19 antibody (#ab52625, Abcam) of 1:1000. Heat-induced antigen retrieval was performed using citrate buffer (pH 6.0) at 95 °C for 20 min before anti-pHH3 antibody staining. Following antigen retrieval, the sections underwent the same blocking, incubation, and visualization procedures as those used for PTK7 staining, ensuring accurate representation of protein expression levels in the tumor samples.

### Pharmacokinetics study of Sgc8c-M

Different doses of Sgc8c-M were injected into female BALB/c nude mice, male Sprague-Dawley (SD) rats, and male cynomolgus monkeys via i.v. injection at a single dose (3.5, 7, and 14 mg/kg for mice; 3.5 and 7 mg/kg for rats; and 1.8 and 3.6 mg/kg for cynomolgus monkeys). Whole blood and tissue samples were subsequently harvested at certain time points.

#### Plasma and tissue sample preparation

Whole blood samples in heparin sodium tubes were centrifuged at 4000 rpm for 10 min at 4 °C, and supernatant plasma was collected and stored at −80 °C for further analysis. Tissue samples were weighed and homogenized in RIPA lysis buffer, using a dilution factor of 5 for the heart, liver, spleen, lung, kidney, small intestine, large intestine, stomach, bladder, and tumor tissues. These tissues were homogenized using a TissueLyser II (QIAGEN) at a frequency of 30 Hz for 30 s, followed by a 30-s ice cooldown. The homogenization operation was repeated twice, and, finally, the tissue homogenate was incubated at 4 °C for 2 h for equilibrium.

#### LC-MS/MS quantification of MMAE

The pharmacokinetics of the MMAE fraction of Sgc8c-M was determined by the LC-MS/MS method using liquid chromatography coupled with a triple quadrupole mass spectrometry system (Agilent 6495 C). Chromatographic separation was performed using an ACQUITY Premier BEH C18 column (1.7 μm, 2.1 × 50 mm, Waters); detailed HPLC conditions are provided in Supplementary Table 5. MMAE (#HY-15162, MCE) was quantified by multiple reaction monitoring (MRM) modes, using d8-MMAE (#HY-15162A, MCE) as an internal standard. The MRM transitions for MMAE and d8-MMAE were 718.51 → 686.6/152.1/134.1 and 726.56 → 694.6/152.1/134.1, respectively.

Free MMAE quantification. To quantify free MMAE, 120 μL acetonitrile containing d8-MMAE (5 ng/mL) were added into 30 μL plasma. After vortex and centrifugation at 13,000 rpm for 15 min at 4 °C, the supernatant was collected for analysis by LC-MS/MS.

Total MMAE quantification. To quantify total MMAE (free MMAE + conjugated MMAE), either CTSB (#10483-H08H, Sino Biological) or fresh liver homogenate was used to cleave the VC linker of Sgc8c-M, thus releasing MMAE. Specifically, 1 μL 0.1 mg/mL CTSB or 30 μL fresh liver homogenate were added to 30 μL plasma and tissue homogenate and incubated at 37 °C for 24 h. Subsequently, a 4-fold volume of acetonitrile containing d8-MMAE (5 ng/mL) was added. After vortex and centrifugation at 13,000 rpm for 15 min at 4 °C, the supernatant was collected for total MMAE quantification by LC-MS/MS.

Standard samples for LC-MS/MS quantification were prepared by spiking with known concentrations of MMAE into plasma and tissue matrices, followed by a sample preparation procedure analogous to that described above.

Major PK parameters were calculated from a non-compartmental analysis (PK solver 2.0, an add-in program in Microsoft Excel) of the samples obtained following intravenous bolus input.

### Toxicity and toxicokinetic study of Sgc8c-M in rats

#### Multiple-dose toxicity study of Sgc8c-M

Forty healthy SD rats of both genders (female: 190–230 g, male: 210–280 g) were randomly assigned to four treatment groups (5 rats/gender/group). The rats received intravenous injections of Sgc8c-M at multiple doses (days 1, 5, 9, and 13) of 0 (equal volume of saline), 3.5, 10.5, and 18 mg/kg, respectively. During the administration period, all rats were observed and recorded daily for clinical signs, mortality, body weight, and food intake (measured in grams per cage). Blood samples were sampled 24 h after the last administration (day 14) for assays of blood routine, blood biochemistry, and blood coagulation. After euthanasia, the rats were dissected, and major organs (including the heart, liver, spleen, lungs, kidneys, and other organs) were excised and weighed to obtain both absolute and relative organ weight data. Subsequently, the major organs were fixed in 10% formalin, embedded in paraffin, cut into paraffin sections (5 μm), stained with hematoxylin and eosin (H&E), and finally visualized under a light microscope.

#### Multiple-dose toxicokinetic study of Sgc8c-M

In order to investigate the toxicokinetic profile of Sgc8c-M in rats, three groups of eighteen SD rats of both genders were established as satellite groups. These groups received i.v. injections of Sgc8c-M at multiple doses of 3.5 mg/kg, 10.5 mg/kg, and 18 mg/kg on days 1, 5, 9, and 13, respectively. Following administration, whole blood samples were collected at various time points (0.083, 0.25, 0.5, 1, 2, 4, 6, 8, and 24 h) post-injection on both day 1 and day 13 into heparin sodium tubes. The collected blood samples were then centrifuged at 4000 rpm for 10 min at 4 °C to obtain supernatant plasma. Finally, the concentration of free and total MMAE in rat plasma was quantified by LC-MS/MS as described above.

### Toxicity and toxicokinetic study of Sgc8c-M in cynomolgus monkeys

Sixteen healthy cynomolgus monkeys of both genders (female: 2.45–3.50 kg, male: 3.95–8.00 kg) were randomly divided into four groups (2 cynomolgus monkeys/gender/group). The monkeys received intravenous injection of Sgc8c-M at multiple doses (days 1, 5, 9, and 13) of 0 (equal volume of saline), 1.8 mg/kg, 3.6 mg/kg, and 5.4 mg/kg, respectively. Blood samples were sampled 24 h before the first dose (day 0) and after the last dose (day 14) for the analysis of blood routine, blood biochemistry, and blood coagulation. During the administration period, all cynomolgus monkeys were monitored daily for clinical signs, mortality, body temperature, body weight, and food intake. For toxicokinetic analysis, blood samples were collected into heparin sodium tubes at nine time points (0.083, 0.25, 0.5, 1, 2, 4, 6, 8, and 24 h) following the first dose on day 1 and the final dose on day 13. The collected blood samples were then centrifuged at 4000 rpm for 10 min at 4 °C to obtain supernatant plasma. The concentration of MMAE was quantified using LC-MS/MS as previously detailed.

The pharmacokinetic, toxicokinetic, and toxicity studies of Sgc8c-M in rats and cynomolgus monkeys were performed by a contract research organization, Frontage.

### Statistical analysis

All variables were presented as mean ± SEM with the exception of PK and TK parameters, which were expressed as mean ± SD. The number of replicates and the statistical analysis for each experiment are indicated in the corresponding figure legends. All statistical analysis was perfumed using GraphPad Prism 8.0 (GraphPad Software, USA). One-way ANOVA or t tests were performed in statistical evaluation. A p-value < 0.05 was considered significant.

## Supplementary information

Clean version_SI

## Data Availability

All data supporting the findings of this study are available within the article and its Supplementary Information.
