# Supplementary material for: An aptamer-drug conjugate for promising cancer therapy with comprehensive evaluation from rodents to non-human primates
Source: Signal Transduct Target Ther. 2025 Sep 24;10:316. doi: 10.1038/s41392-025-02399-1 (PMC12457637; doi:10.1038/s41392-025-02399-1)
Supplement: Supplementary file 1 — Clean version_SI [file 41392_2025_2399_MOESM1_ESM.docx]

Supplementary Materials for

An aptamer-drug conjugate for promising cancer therapy with comprehensive evaluation from rodents to non-human primates

Minhui Su, Yuan Liu, Hongxin Lin, Xiaoxing Wang, Danxia Ying, Lizhuan Zhang, Cai Yang, Mengyuan Jiang, Lujuan Xu, Xie Wang, Yang Sun, Haiyan Xu, Ziwen Zhang, Xiaojia Wang, Ting Fu, Sitao Xie, Jiaxuan He^*^, Xiangsheng Liu^*^, Weihong Tan^*^

^*^Correspondence to: tan@him.cas.cn; liuxs@him.cas.cn; hejiaxuan@him.cas.cn.

**This PDF file includes:**

Supplementary Fig. 1 to Supplementary Fig. 25

Supplementary Table 1 to Supplementary Table 7


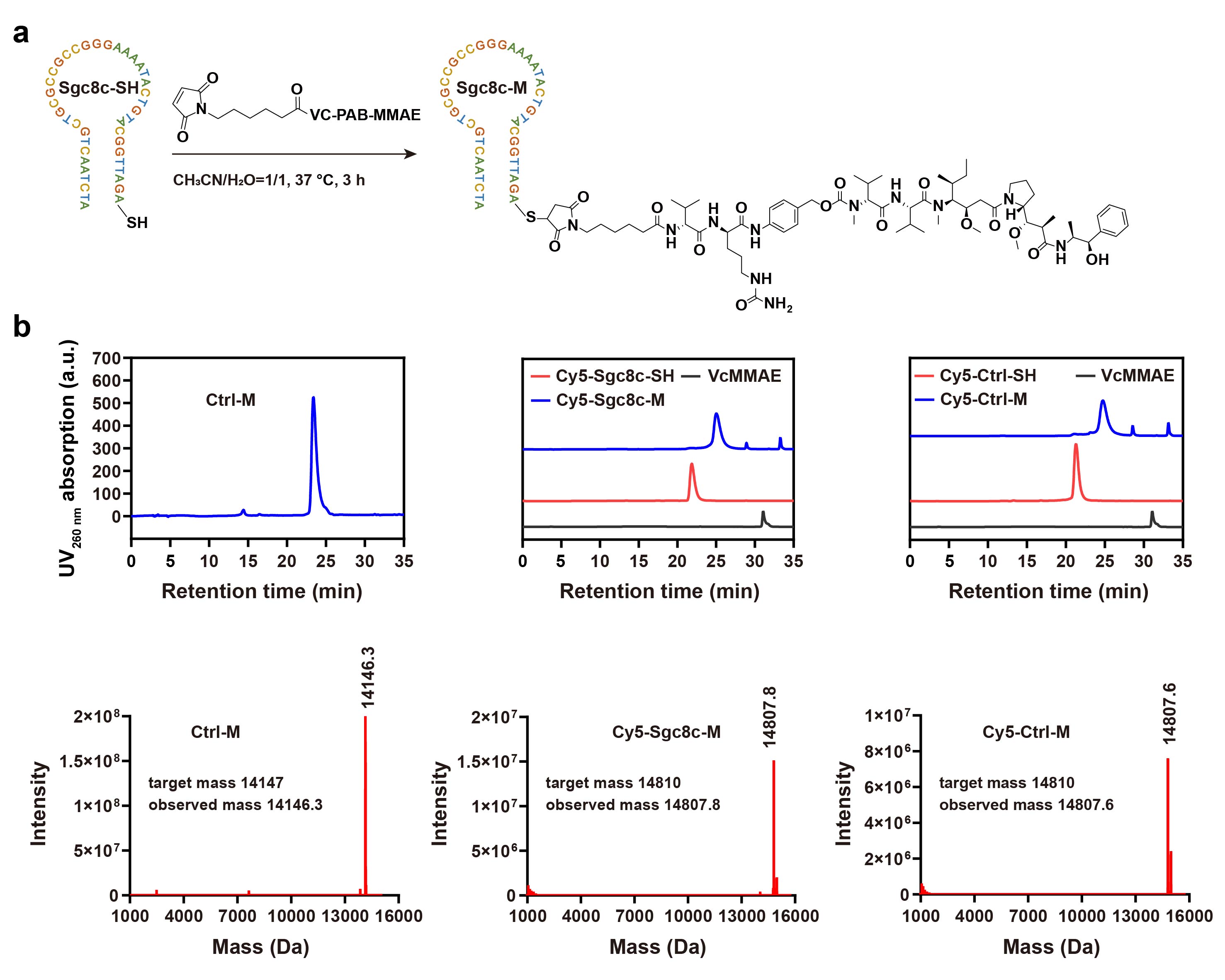


**Supplementary Fig. 1**

**Synthesis route, HPLC, and mass** **characterization of different drug conjugates.** **(a)** Synthesis route of Sgc8c-M. **(b)** HPLC and mass spectrum of Ctrl-M, Cy5-Sgc8c-M, and Cy5-Ctrl-M.

**
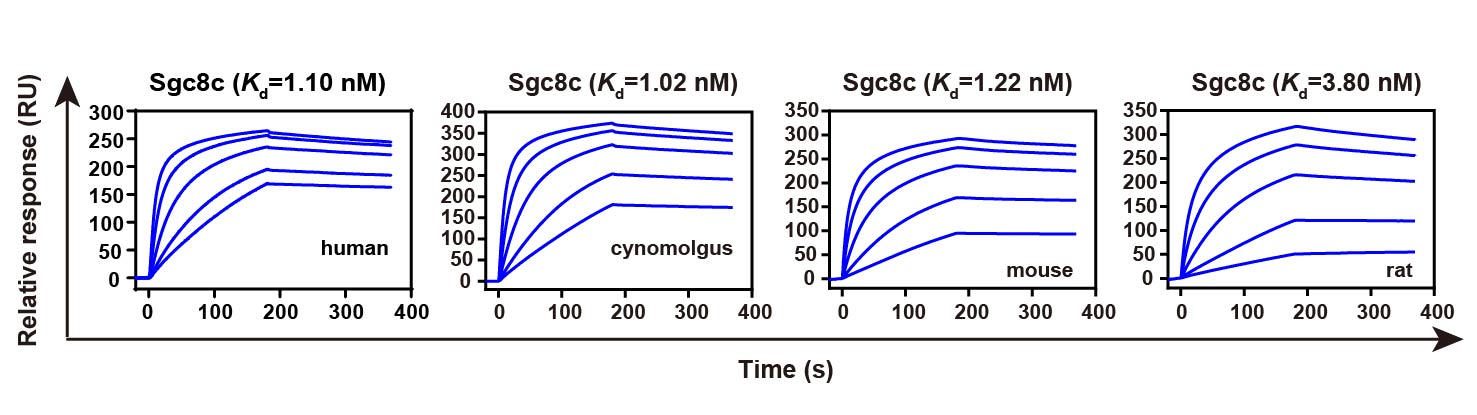
**

**Supplementary Fig. 2**

Surface plasmon resonance (SPR) analysis of Sgc8c for binding to recombinant PTK7 protein of human, cynomolgus monkey, mouse, and rat. The concentrations of Sgc8c from top to bottom are 400, 200, 100, 50, and 25 nM.


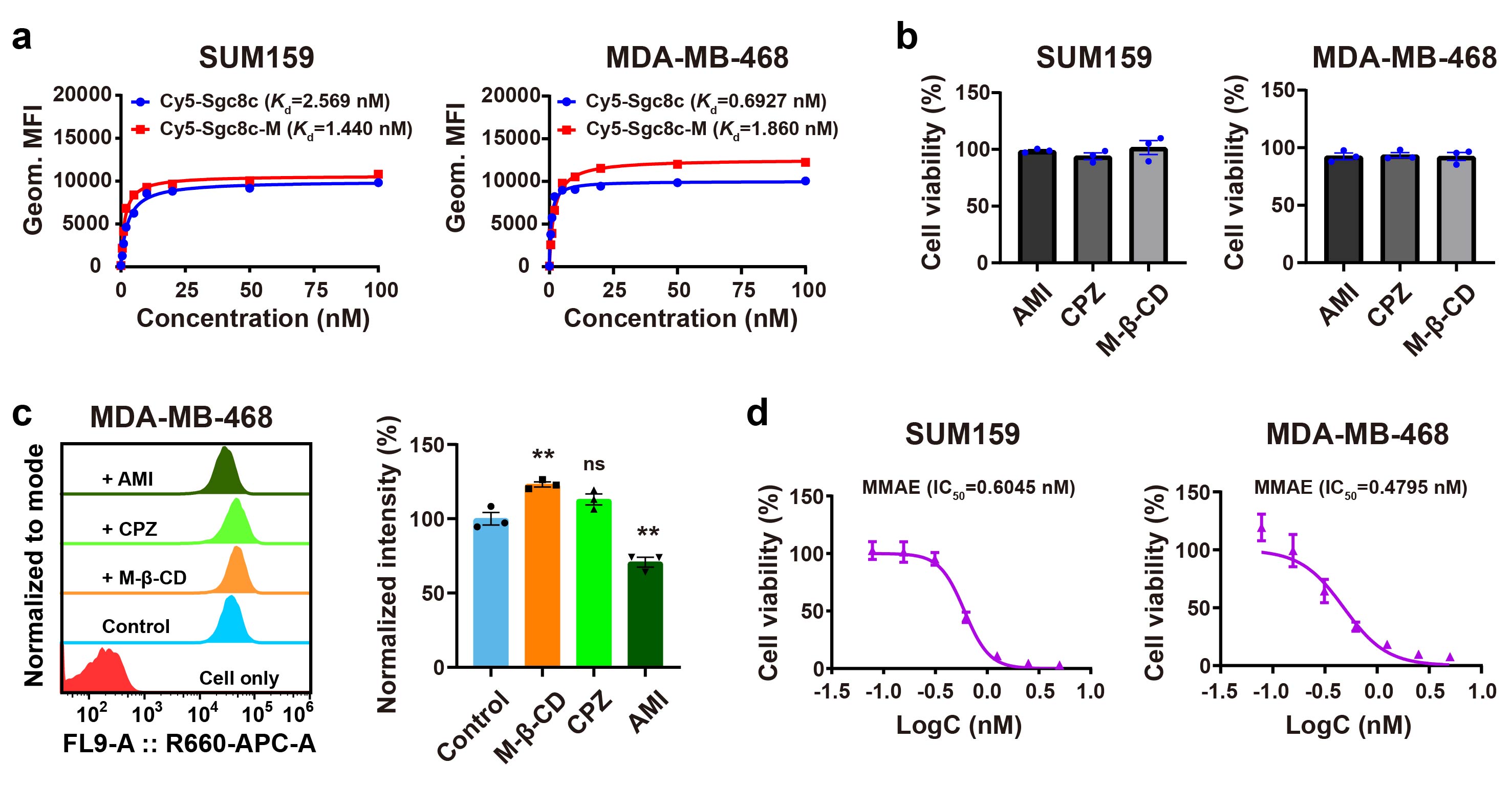


**Supplementary Fig. 3**

(**a**) The binding affinity of Cy5-Sgc8c and Cy5-Sgc8c-M to PTK7-positive SUM159 (*n* = 2) and MDA-MB-468 (*n* = 3) cells was evaluated by flow cytometry. Geom. MFI represents the geometric mean fluorescence intensity. (**b**) Cytotoxicity assays of endocytosis inhibitors at working concentrations in SUM159 and MDA-MB-468 cells for a 2.5-h incubation (*n* = 3). For SUM159 cells, the concentrations of AMI, CPZ, and M-β-CD were 100 μM, 25 μM, and 200 μM, respectively. For MDA-MB-468, the concentrations of AMI, CPZ, and M-β-CD were 50 μM, 6.25 μM, and 40 μM, respectively. (**c**) Endocytic pathway study of Cy5-Sgc8c-M in MDA-MB-468 cells (*n* = 3). M-β-CD (40 μM), CPZ (6.25 μM), and AMI (50 μM) were preincubated with cells for 30 min at 37 °C; then, 400 nM Cy5-Sgc8c-M were added, followed by incubation for another 2 h. Unpaired t test was used in comparison to control, ***P* < 0.01, ns: not significant. (**d**) Cytotoxicity of MMAE in SUM159 and MDA-MB-468 cells for a 72-h incubation (*n* = 3). Data are presented as mean ± SEM.

**
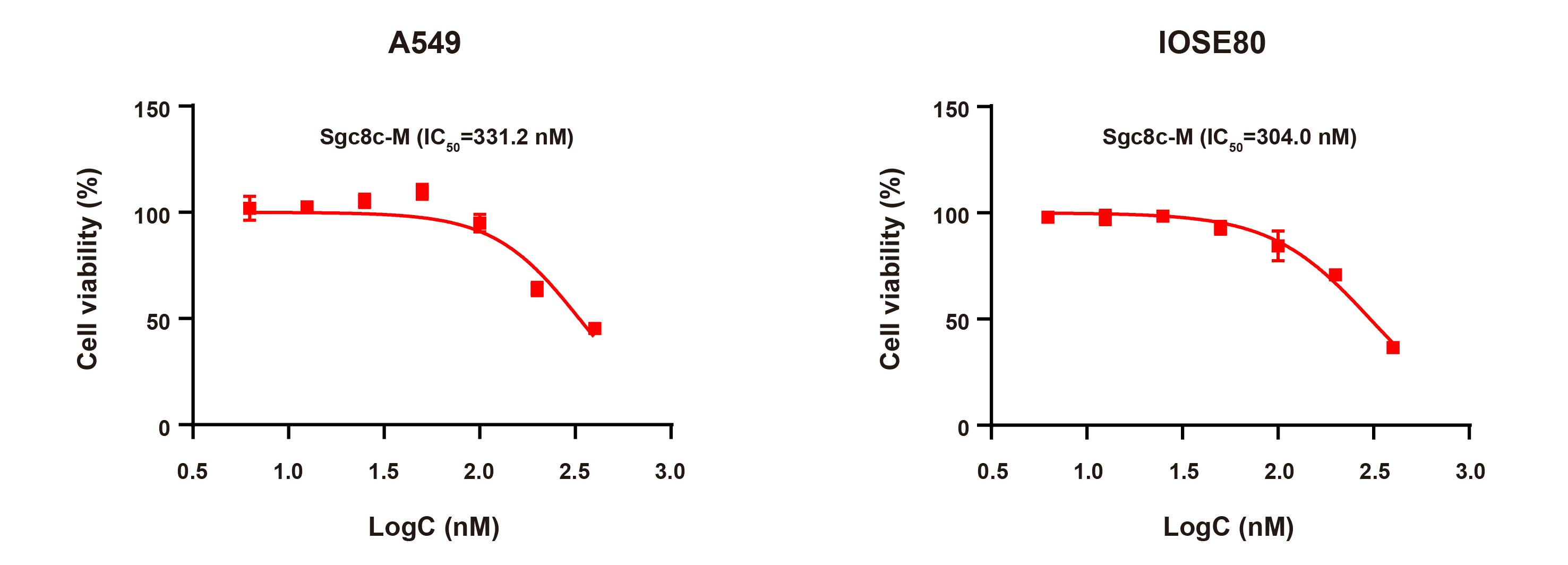
**

**Supplementary Fig. 4**

*In vitro* cytotoxicity of Sgc8c-M against A549 cancer cells and human normal ovarian epithelial cells IOSE80 after a 72-h incubation. Data are presented as mean ± SEM (*n* = 3).


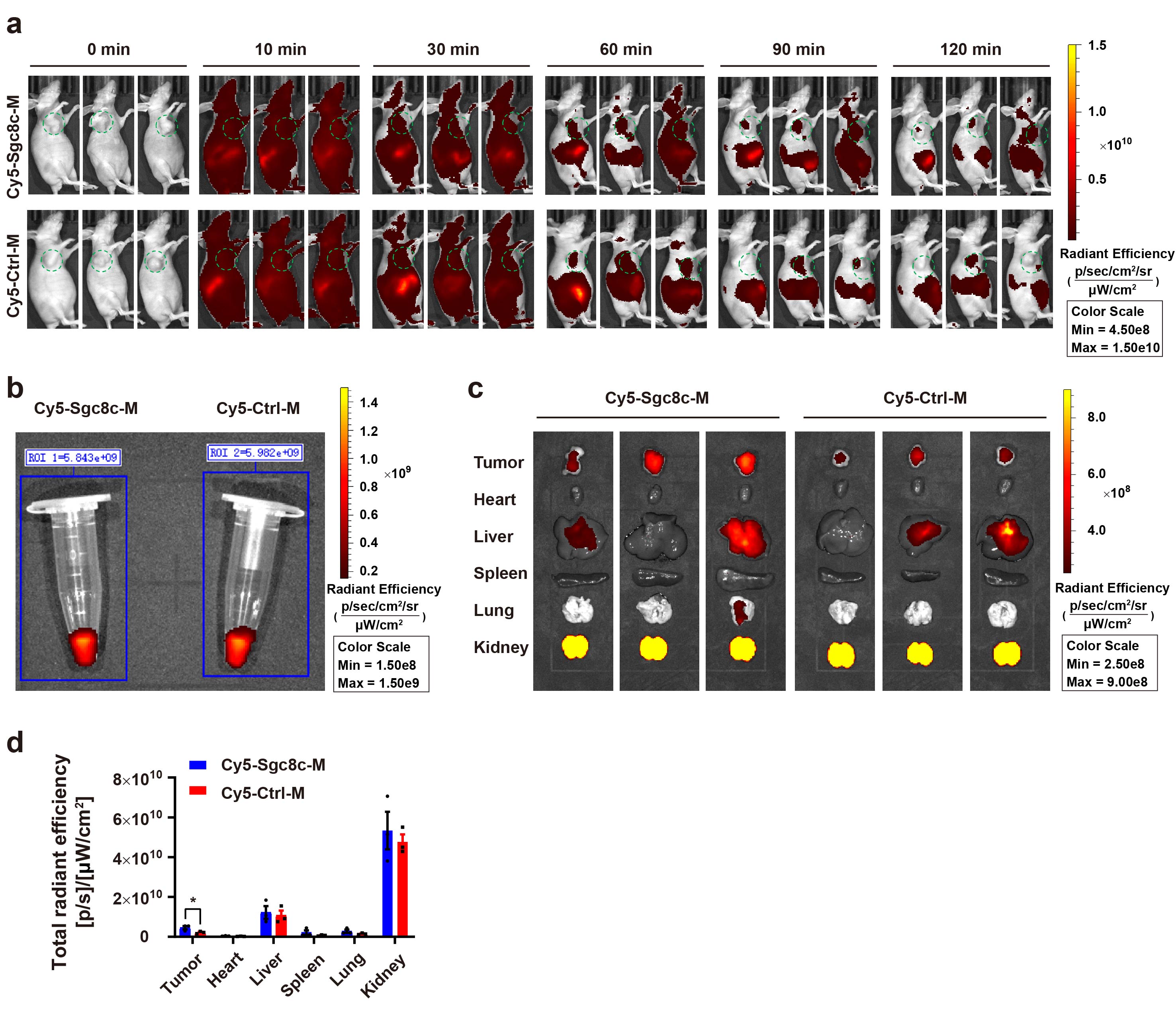


**Supplementary Fig. 5**

***In vivo* fluorescence imaging of Cy5-labeled Sgc8c-M and Ctrl-M on MDA-MB-468 tumor-bearing mice.** **(a)** The distribution of Cy5-Sgc8c-M and Cy5-Ctrl-M (10 μM, 200 μL) in MDA-MB-468 tumor-bearing mice within 2 h. **(b)** Fluorescence imaging of Cy5-Sgc8c-M and Cy5-Ctrl-M in tubes after 100 times dilution to ensure consistent fluorescence when injected intravenously into mice. **(c)** Fluorescence imaging of Cy5-Sgc8c-M and Cy5-Ctrl-M in MDA-MB-468 tumors and major organs at 2 h post-intravenous injection. **(d)** Quantification of fluorescence intensity of Cy5-Sgc8c-M and Cy5-Ctrl-M in MDA-MB-468 tumors and major organs. Data are presented as mean ± SEM (*n* = 3). Paired t test was used, **P* < 0.05.


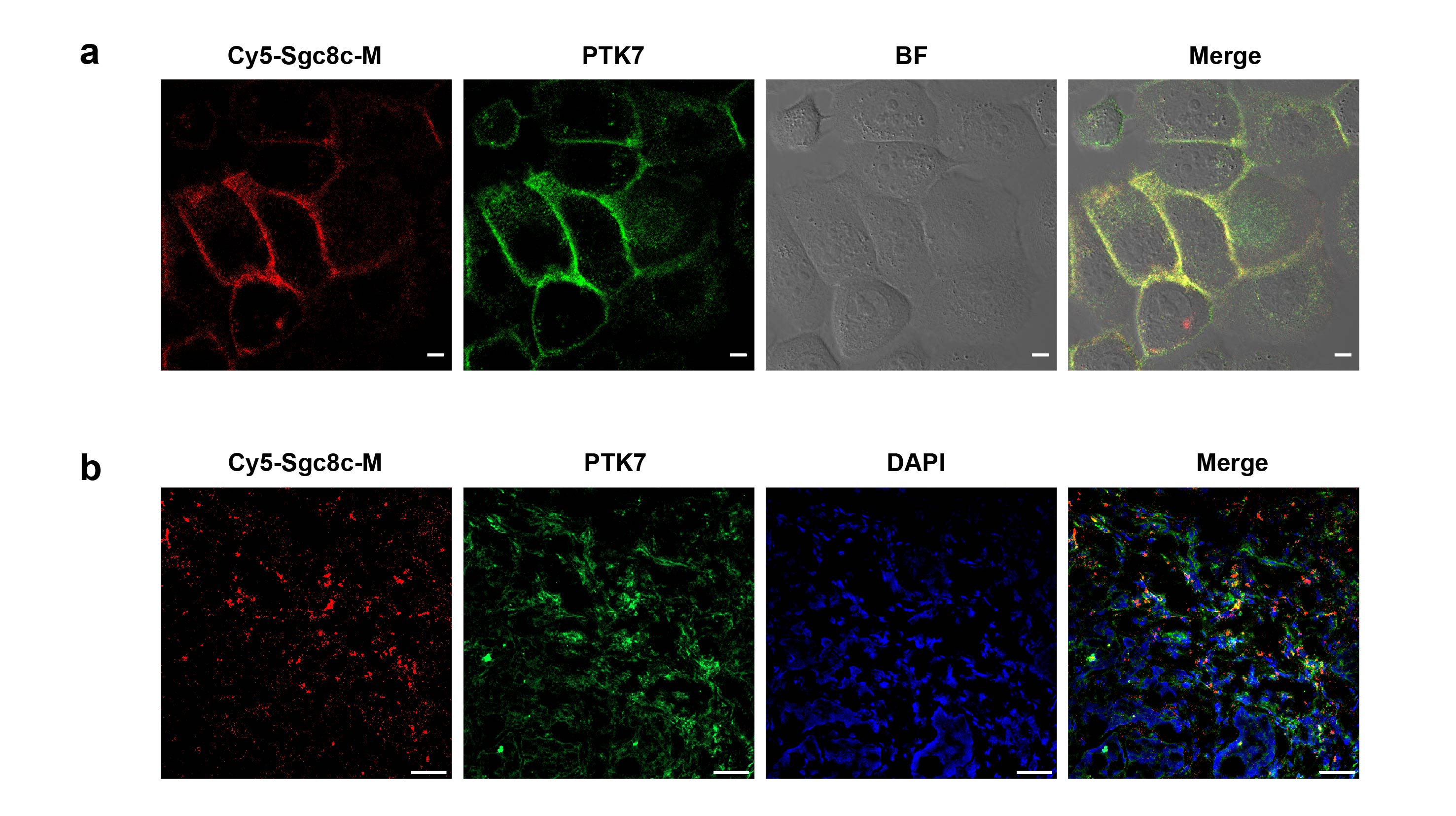


**Supplementary Fig. 6**

**Colocalization studies of Sgc8c-M with PTK7 in SUM159 cells and tumors. (a)** Representative microscopy images of SUM159 cells treated with Cy5-Sgc8c-M followed by Alexa Fluor 488-labeled PTK7 antibody labeling. The colocalization of Cy5-Sgc8c-M (red) and Alexa Fluor 488-labeled PTK7 (green) is indicated by yellow in the merged image. Scale bars, 10 µm. **(b)** Representative fluorescence micrographs of Cy5-Sgc8c-M and PTK7 immunofluorescence of SUM159 tumor sections 2 h after intravenous injection of 7 mg/kg Cy5-Sgc8c-M. The colocalization of Cy5-Sgc8c-M (red) and Cy3-labeled PTK7 (green) is indicated by yellow in the merged image. Nuclei stained by DAPI (blue). Scale bars, 50 µm.

**
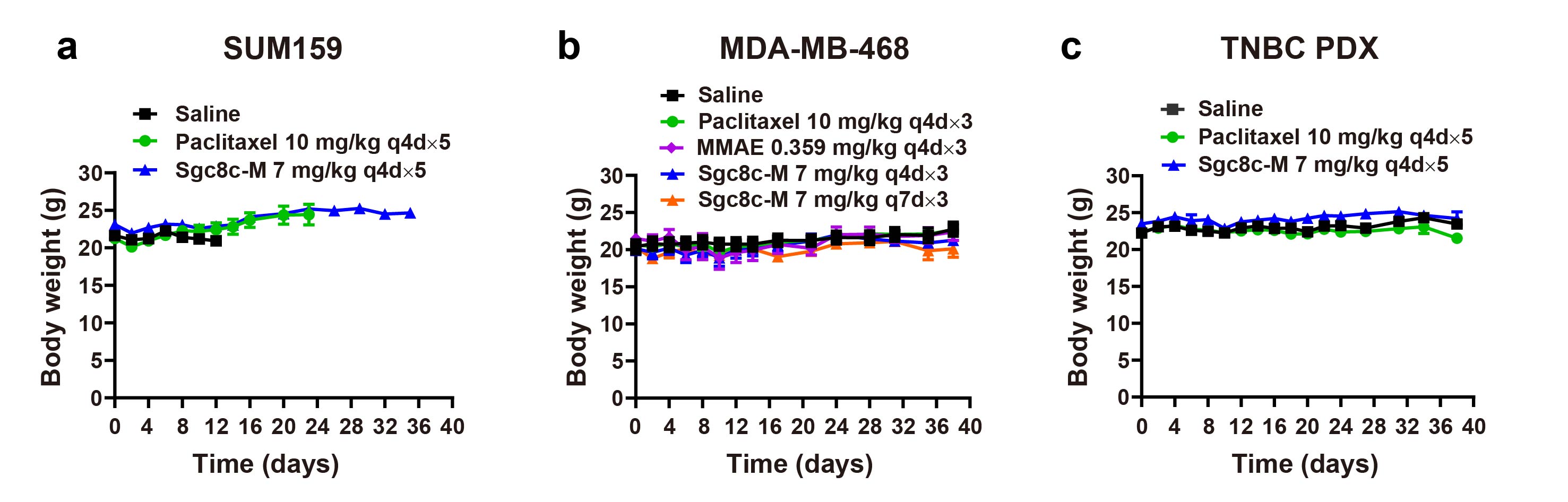
**

**Supplementary Fig. 7**

**Body weights of SUM159, MDA-MB-468, and TNBC PDX tumor-bearing** **mice in antitumor effect study of Sgc8c-M.** **a**, **b**, and **c**, respectively, represent the body weights of mice in Fig. 2c, d, and f of the main manuscript. Error bars in **a** and **b** indicate SEM of *n* = 4, and error bars in **c** indicate SEM of *n* = 5.


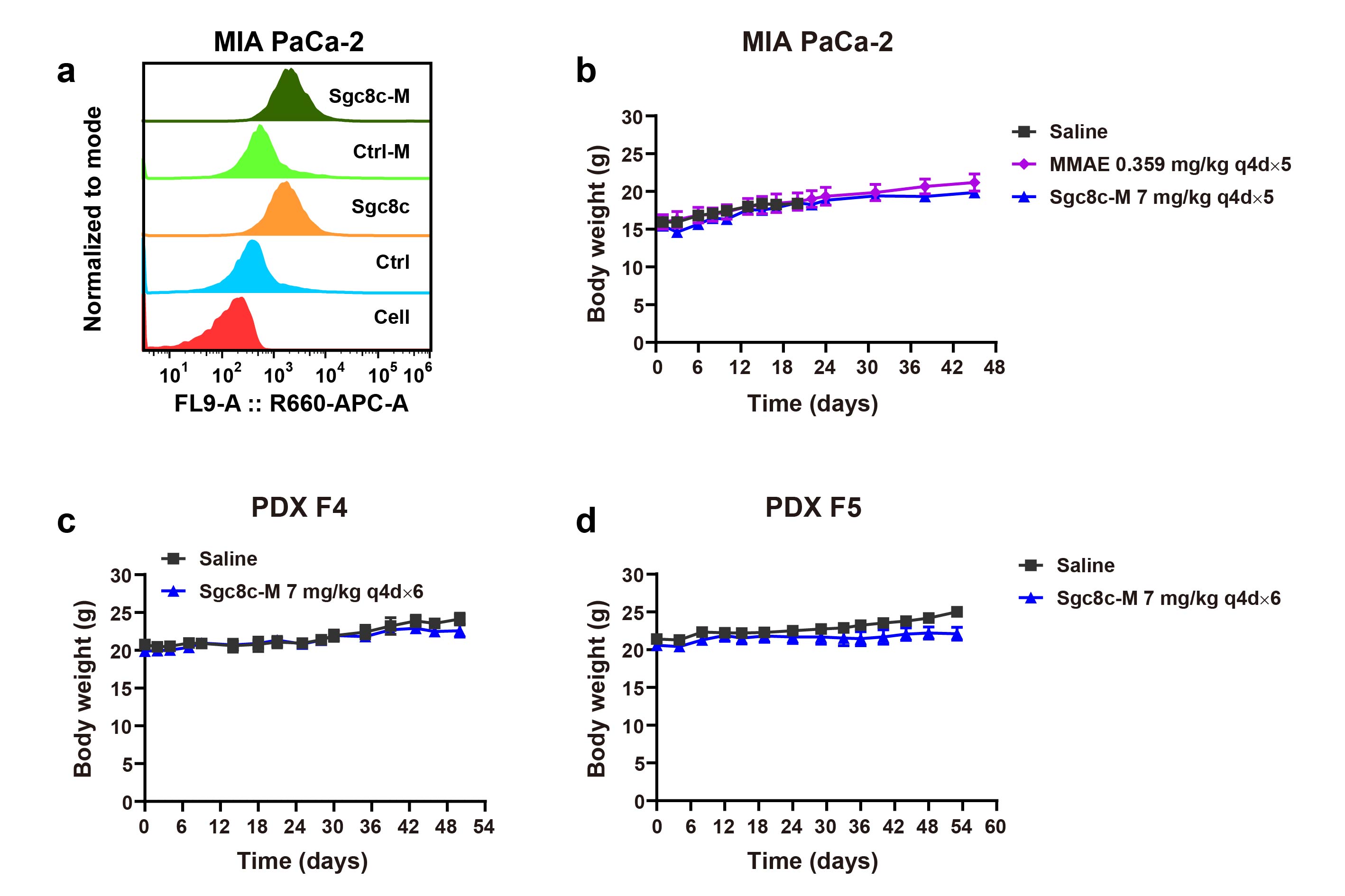


**Supplementary Fig. 8**

**(a)** Cell binding assay of Cy5-labeled Ctrl, Sgc8c, Ctrl-M, and Sgc8c-M (250 nM) to MIA PaCa-2 cells by flow cytometry. **(b, c, and d)** Body weights of MIA PaCa-2, PDX F4 and PDX F5 tumor-bearing mice in antitumor effects study of Sgc8c-M. **b**, **c**, and **d** represent body weights of mice in Fig. 3b, e, and f, respectively. Data are presented as mean ± SEM (*n* = 5).


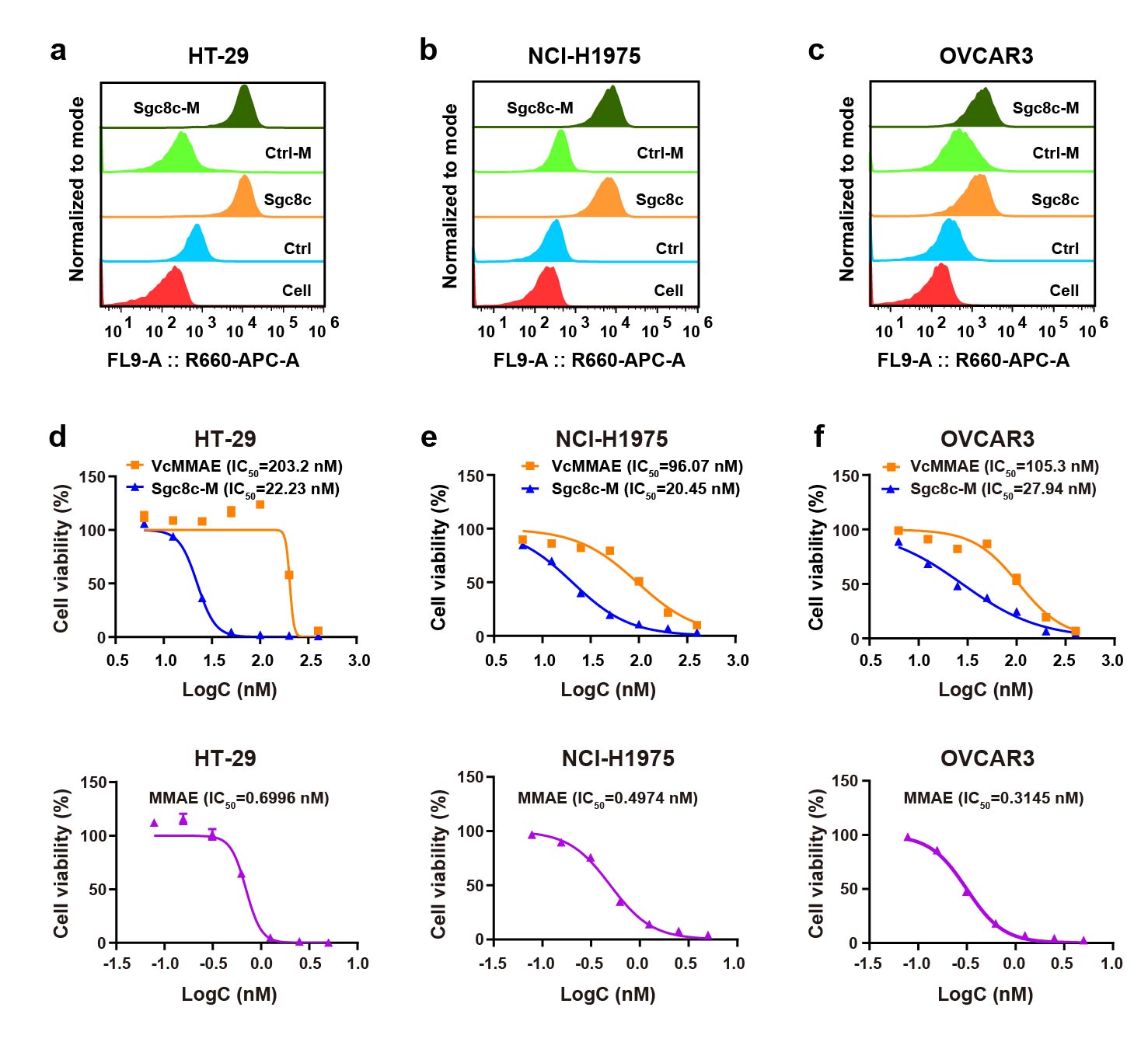


**Supplementary Fig. 9**

**(a-c)** Cell binding assay of Cy5-labeled Ctrl, Sgc8c, Ctrl-M, and Sgc8c-M (250 nM) to HT-29, NCI-H1975, and OVCAR3 cells by flow cytometry. **(d-f)** Cytotoxicity analysis of VcMMAE, Sgc8c-M, and MMAE against HT-29, NCI-H1975, and OVCAR3 cells for 72 h. Data are presented as mean ± SEM (*n* = 3).


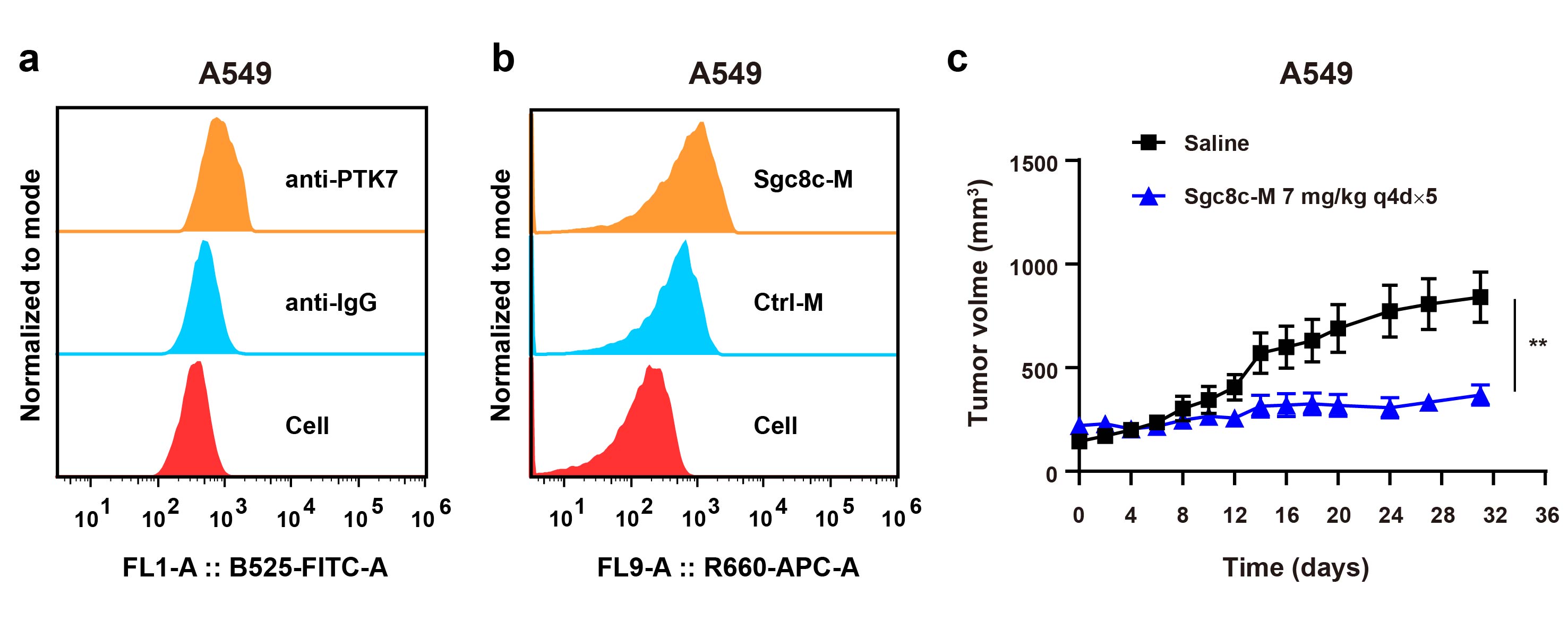


**Supplementary Fig. 10**

**The antitumor effect of Sgc8c-M on PTK7-negative A549 model.** **(a)** The PTK7 expression on A549 cells evaluated by flow cytometry. **(b)** Cell binding of Cy5-labled Ctrl-M and Sgc8c-M (250 nM) to A549 cells. **(c)** Antitumor effects of Sgc8c-M for A549 *in vivo*. A549 tumor-bearing mice were dosed by i.v. every four days for 5 cycles with 7 mg/kg Sgc8c-M. Data are presented as mean ± SEM (*n* = 5). To compare the difference, unpaired t test was used, ***P* < 0.01.

**
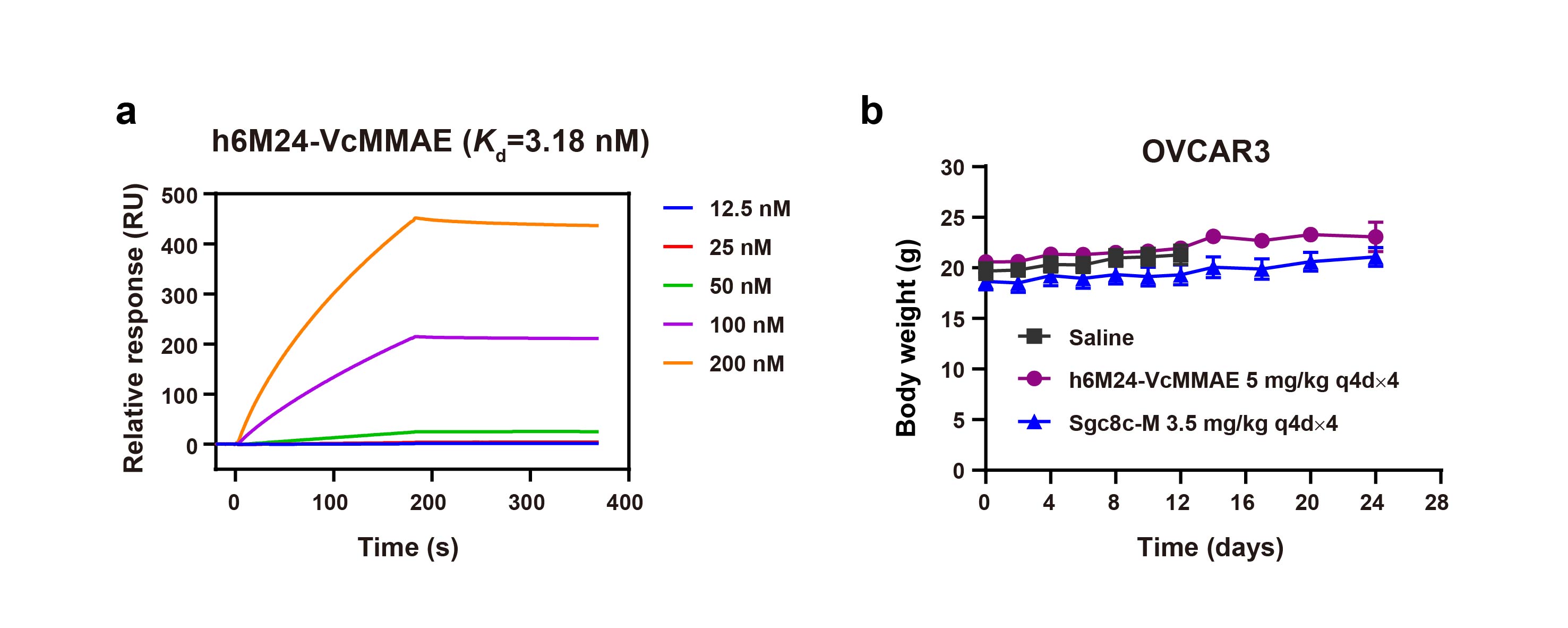
**

**Supplementary Fig. 11**

***In vitro* and *in vivo* characterization for PTK7-targeted ADC h6M24-VcMMAE (DAR4). (a)** SPR analysis of h6M24-VcMMAE for binding to recombinant PTK7 protein of human. **(b)** Body weights of OVCAR3 tumor-bearing mice in the comparison of the antitumor effects of Sgc8c-M and h6M24-VcMMAE in Fig. 4d. Data are presented as mean ± SEM (*n* = 5).


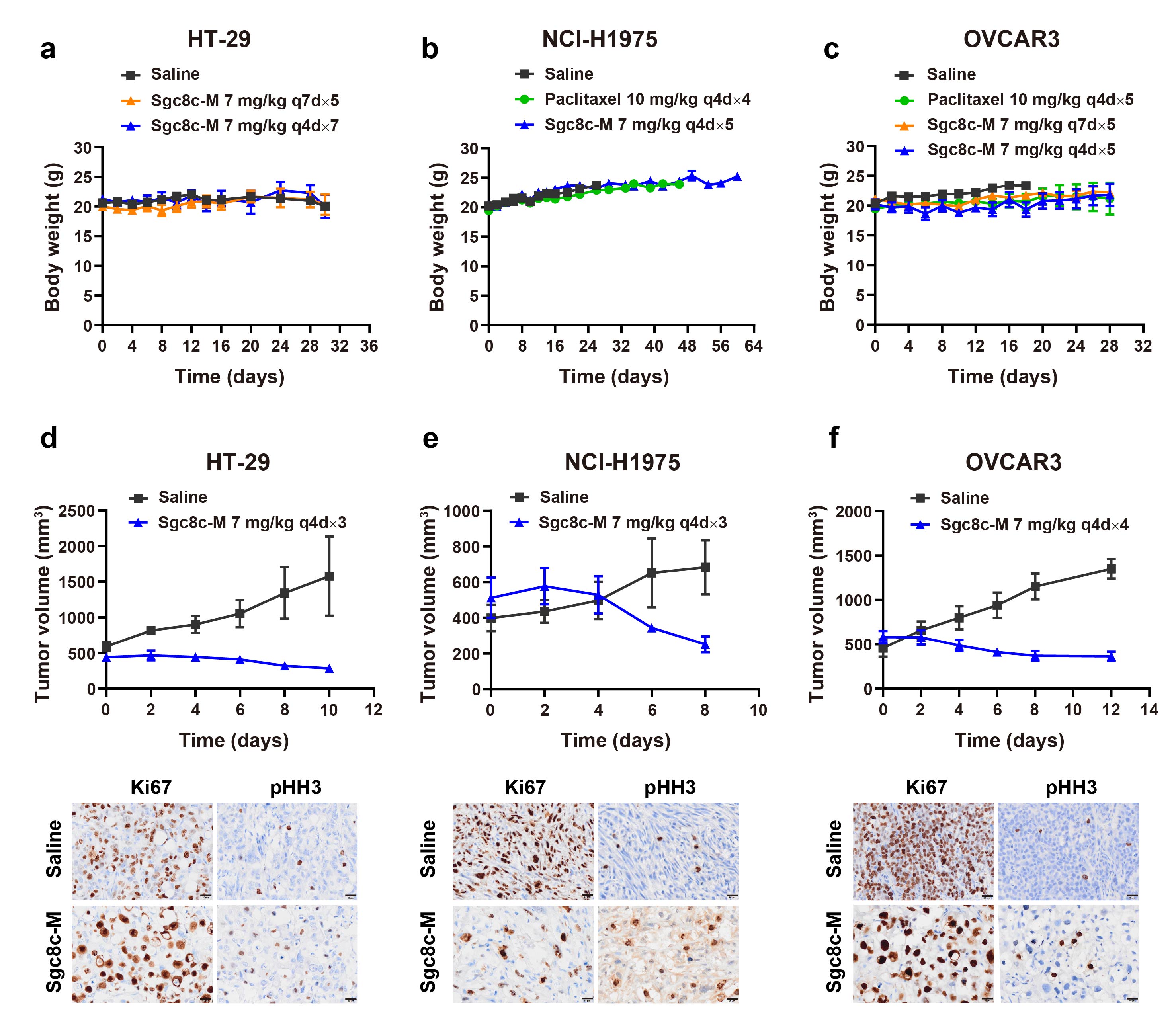


**Supplementary Fig. 12**

**(a-c)** Body weights of HT-29 (*n* = 4), NCI-H1975 (*n* = 3), and OVCAR3 (*n* = 3) tumor-bearing mice in antitumor effect study of Sgc8c-M in Fig. 4. **(d-f)** Evaluation of antitumor and tumor-killing effects study of Sgc8c-M on large tumors (~500 mm^3^) in HT-29 (*n* = 2 for Saline, *n* = 3 for Sgc8c-M), NCI-H1975 (*n* = 2), and OVCAR3 (*n* = 3) models. IHC characterization of Ki67 and pHH3 was performed at 48 - 72 h (72 h for HT-29 and NCI-H1975, and 48 h for OVCAR3) at the end of administration. Scale bars, 20 μm. Data are presented as mean ± SEM.


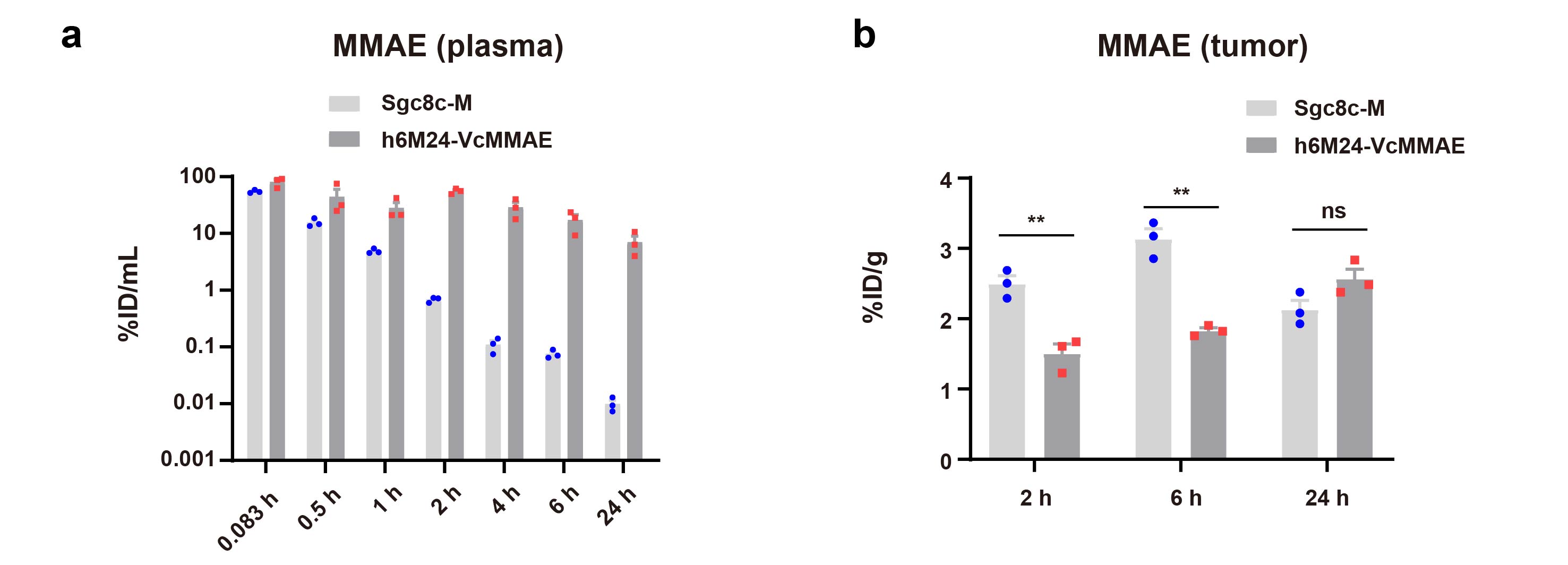


**Supplementary Fig. 13**

**The pharmacokinetic comparison of Sgc8c-M and h6M24-VcMMAE in the OVCAR3 model. (a)** %ID/mL of total MMAE in the plasma of OVCAR3 tumor-bearing mice after intravenous administration of 7 mg/kg Sgc8c-M or 5 mg/kg h6M24-VcMMAE. **(b)** %ID/g of total MMAE in OVCAR3 tumors at 2, 6, and 24 h after a single dose of 7 mg/kg Sgc8c-M or 5 mg/kg h6M24-VcMMAE. Data are presented as mean ± SEM (*n* = 3). To compare the difference, unpaired t test was used, ***P* < 0.01, ns: not significant.


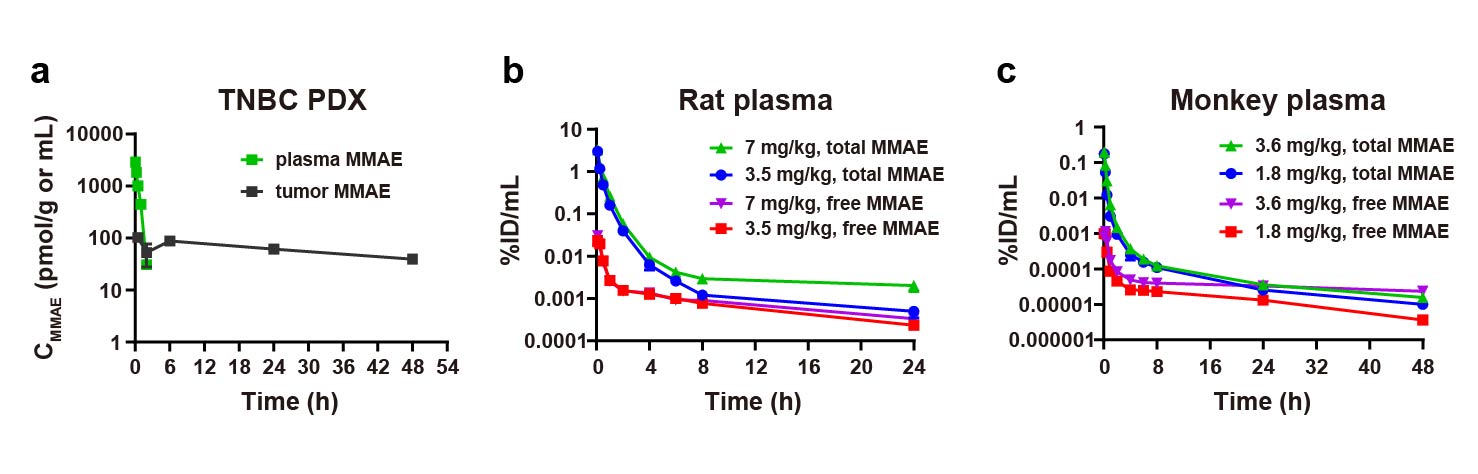


**Supplementary Fig. 14**

**(a)** Pharmacokinetic (PK) profiles of total MMAE in plasma and tumors after administration of 7 mg/kg Sgc8c-M to TNBC PDX tumor-bearing mice. %ID/mL of free and total MMAE in plasma of rats **(b)** and cynomolgus monkeys **(c)**. Data are presented as mean ± SEM (*n* = 3).


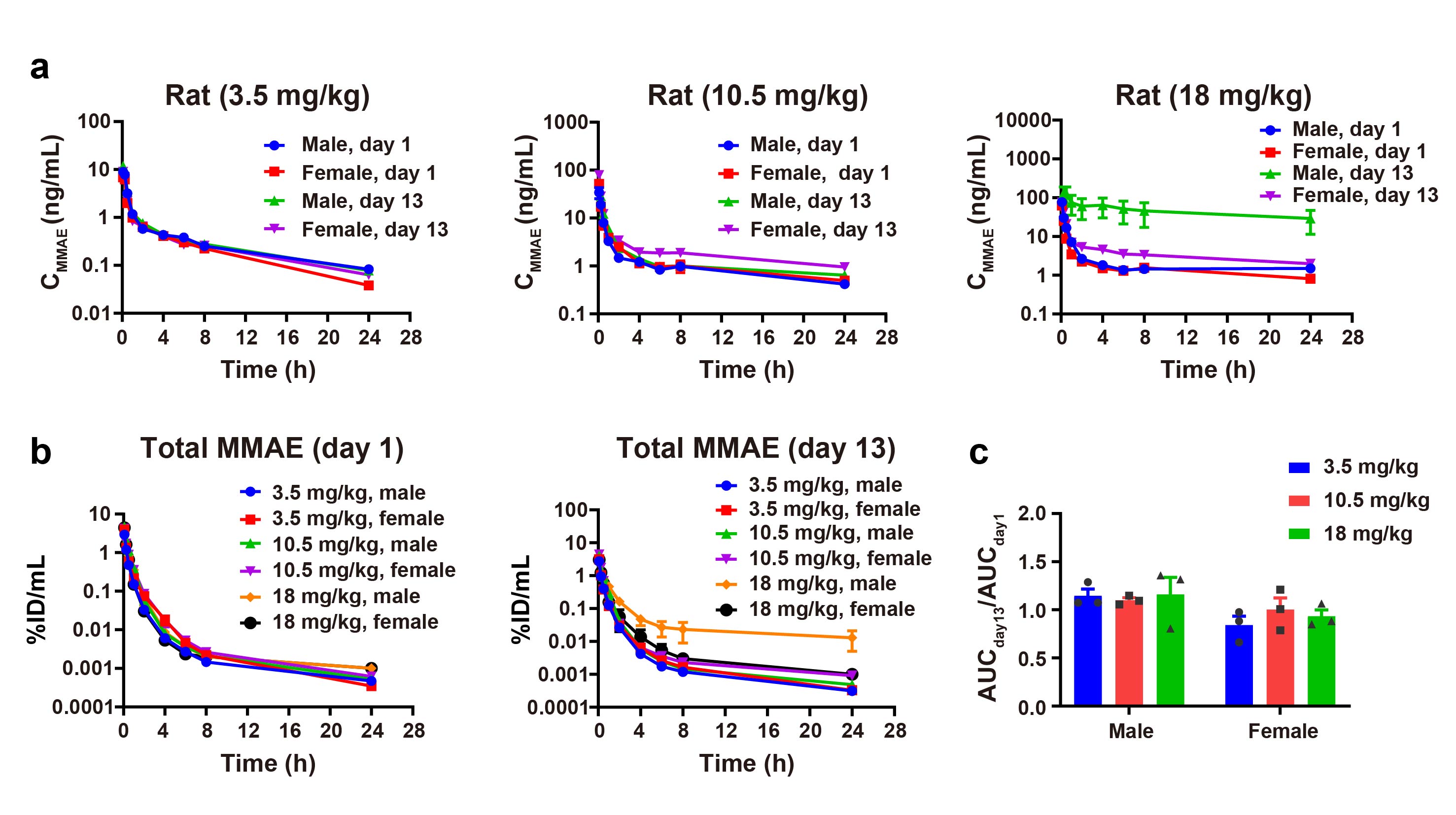


**Supplementary Fig. 15**

**Toxicokinetic (TK) study of Sgc8c-M in rats. (a)** Plasma concentration-time curve of free MMAE in rats after intravenous injection of Sgc8c-M at three doses (3.5, 10.5, and 18 mg/kg) on day 1 and day 13 during the repeated-dose TK study. **(b)** %ID/mL of total MMAE on day 1 and day 13 in rats. **(c)** AUC ratio of total MMAE on day 13 and day 1 in rats. Data are presented as mean ± SEM (*n* = 3).


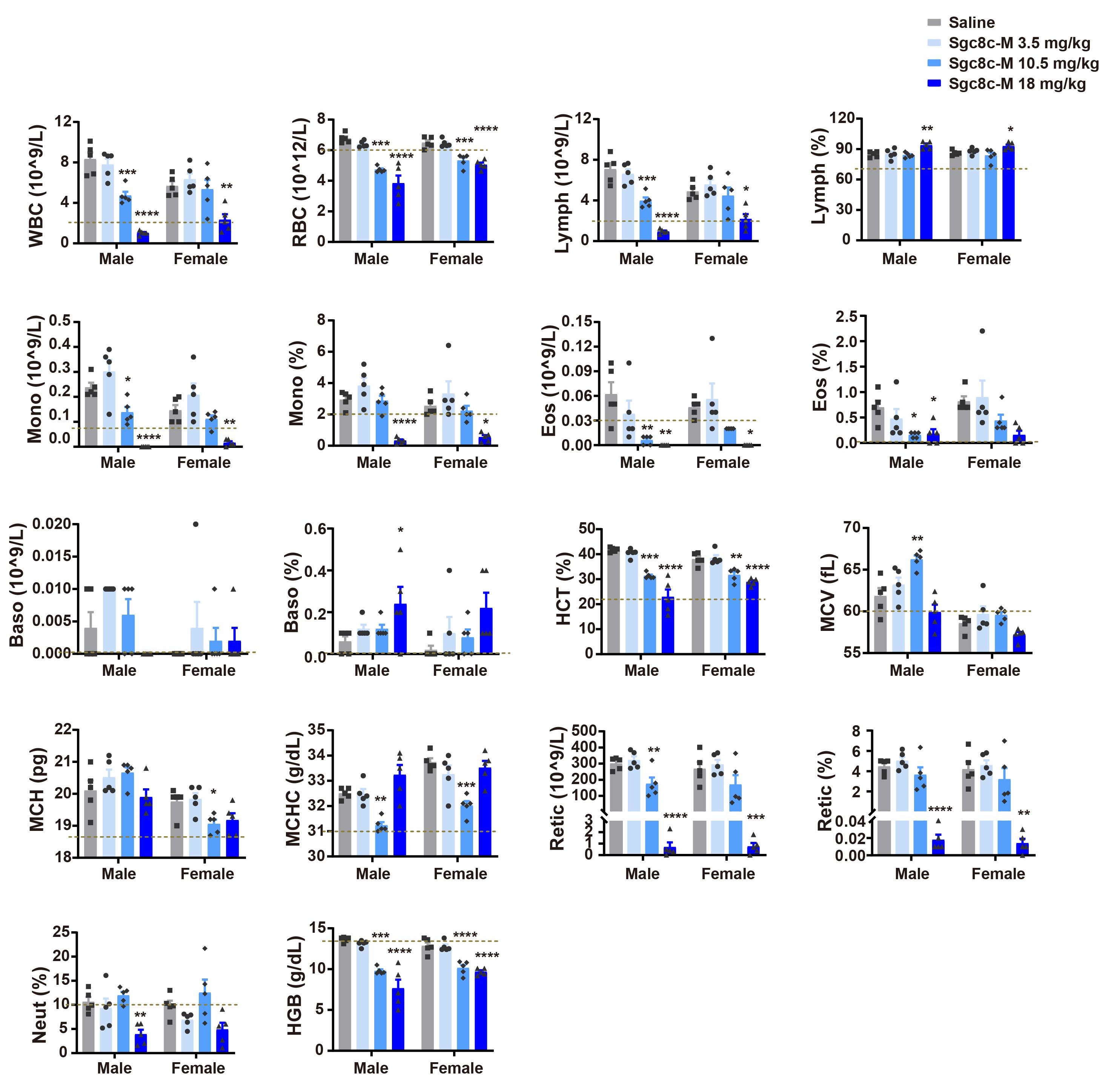


**Supplementary Fig. 16**

Blood routine parameters in rats on day 14 after multiple intravenous injections of 3.5, 10.5, and 18 mg/kg Sgc8c-M. The results shown here are from the same experiment as that shown in Fig. 6d. Data are presented as the mean ± SEM (*n* = 5). One-way ANOVA was used, **P* < 0.05, ***P* < 0.01, ****P* < 0.001, *****P* < 0.0001.


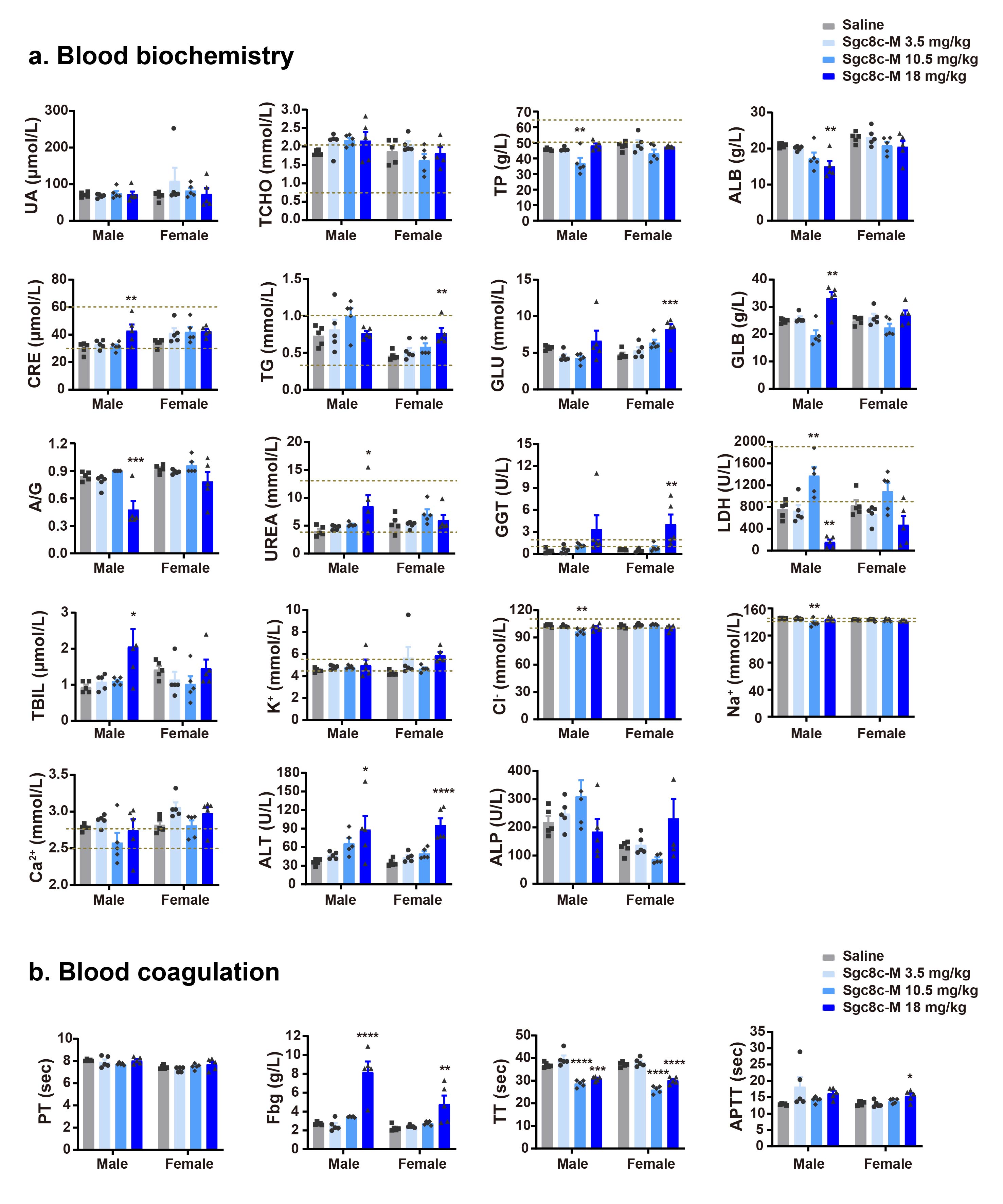


**Supplementary Fig. 17**

Blood biochemistry **(a)** and blood coagulation parameters **(b)** in rats on day 14 after multiple intravenous injections of 3.5, 10.5, and 18 mg/kg Sgc8c-M. The results shown here are from the same experiment as that shown in Fig. 6d. Data are presented as the mean ± SEM (*n* = 5). One-way ANOVA was used, **P* < 0.05, ***P* < 0.01, ****P* < 0.001, *****P* < 0.0001.


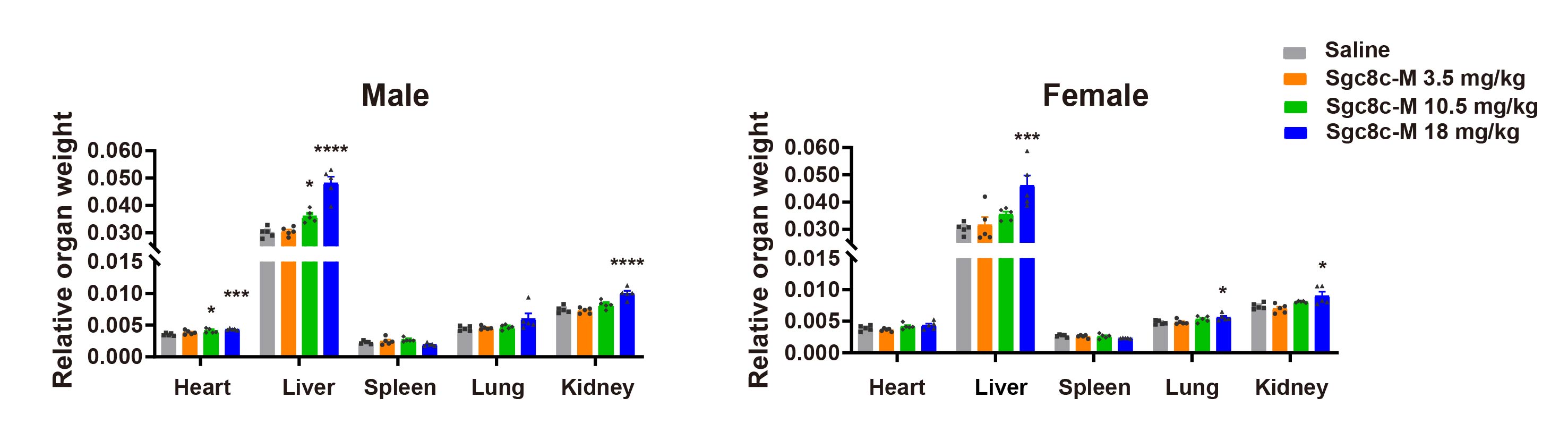


**Supplementary Fig. 18**

Relative organ weights of male and female rats in the repeated-dose toxicity study of Sgc8c-M. Data are presented as the mean ± SEM (*n* = 5). One-way ANOVA was used, **P* < 0.05, ****P* < 0.001, *****P* < 0.0001.


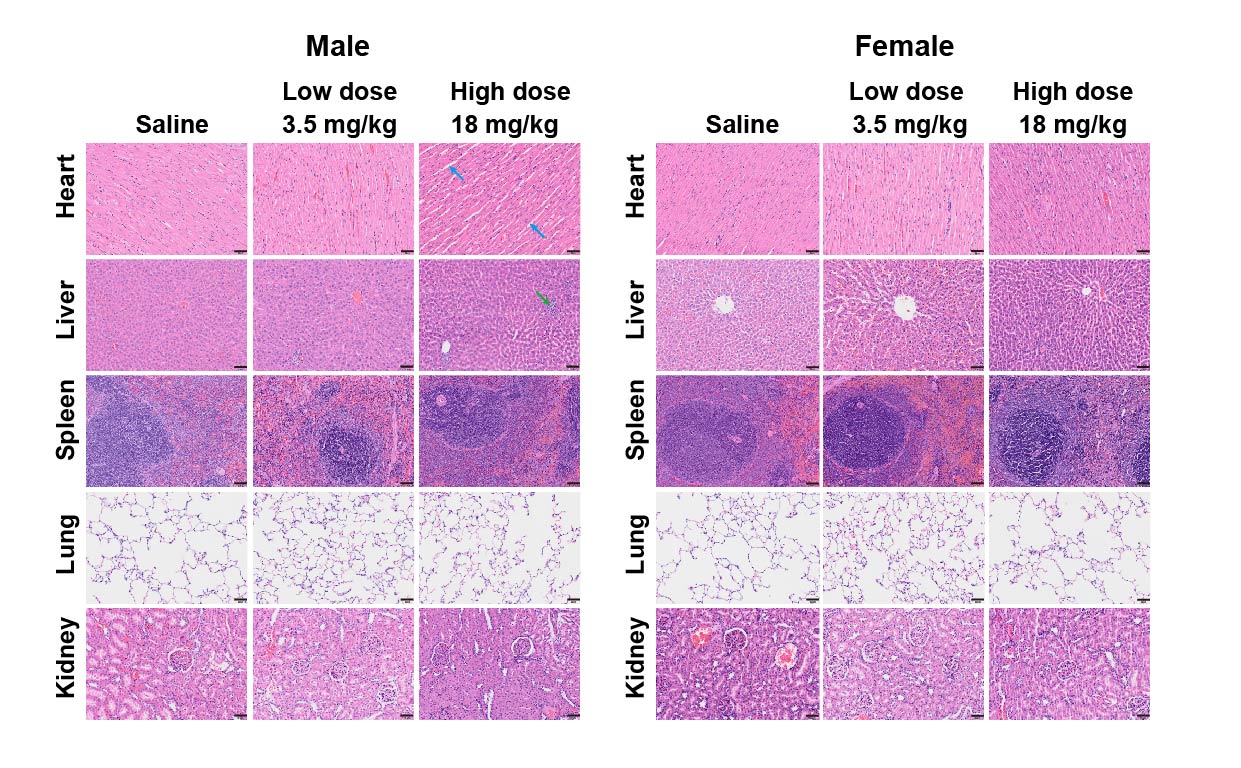


**Supplementary Fig. 19**

H&E-stained major organs of male and female rats treated with 3.5 mg/kg (low dose) and 18 mg/kg (high dose) of Sgc8c-M in the repeated-dose toxicity study. Blue arrows show atrophy of myocardial fibers with widened gaps. Green arrows show sites of inflammatory cell infiltration. Scale bars, 50 μm.


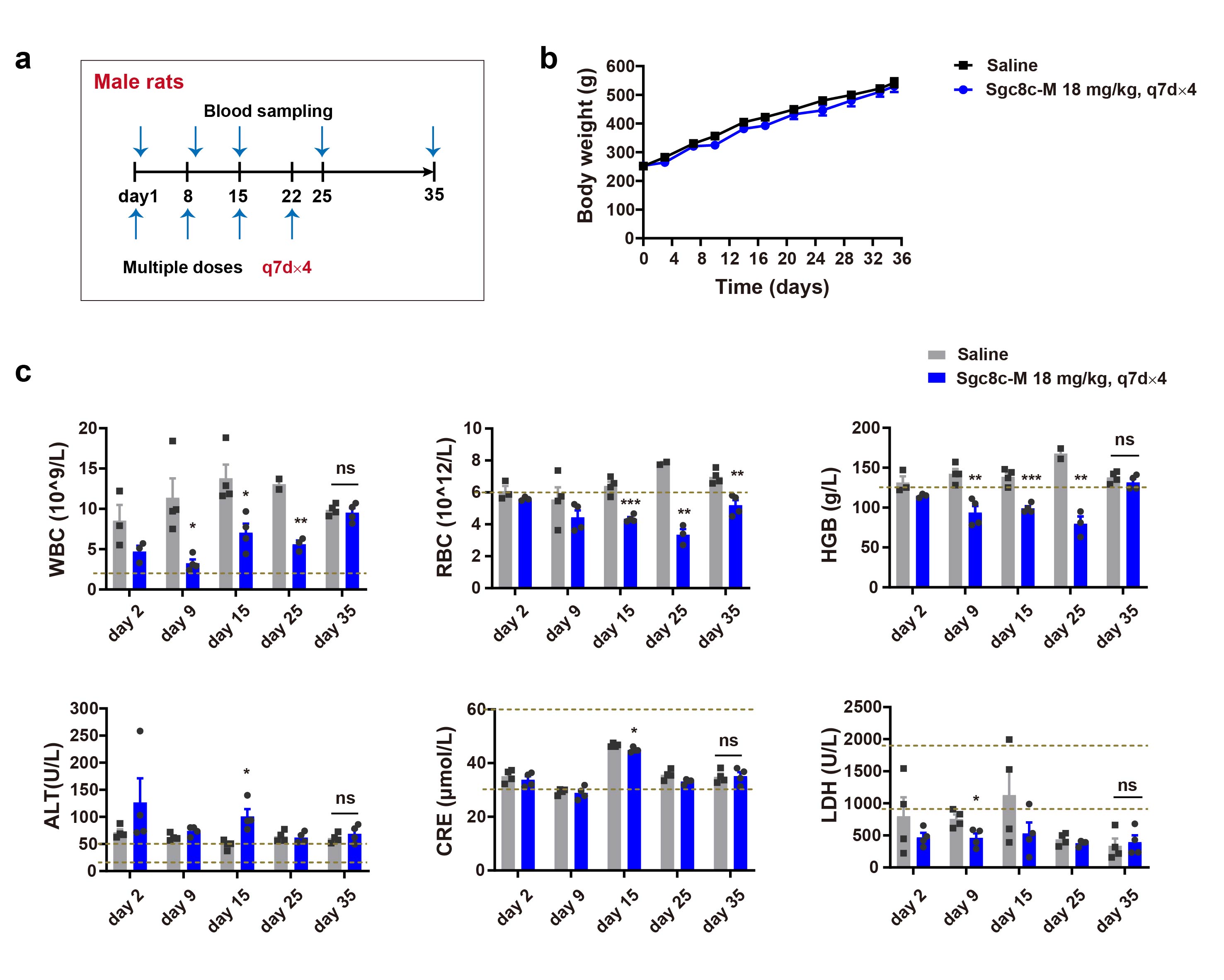


**Supplementary Fig. 20**

**(a)** Treatment regimen and blood sampling schedule in male rats with the dosing interval of the 18 mg/kg Sgc8c-M extended to q7d×4. **(b)** Body weight monitor and **(c)** blood routine and blood biochemistry parameters measurement after the treatment. The results shown here are from the same experiment as that shown in Fig. 6f. Data are presented as the mean ± SEM (*n* = 4). Unpaired t test was used, **P* < 0.05, ***P* < 0.01, ****P* < 0.001, ns: not significant.


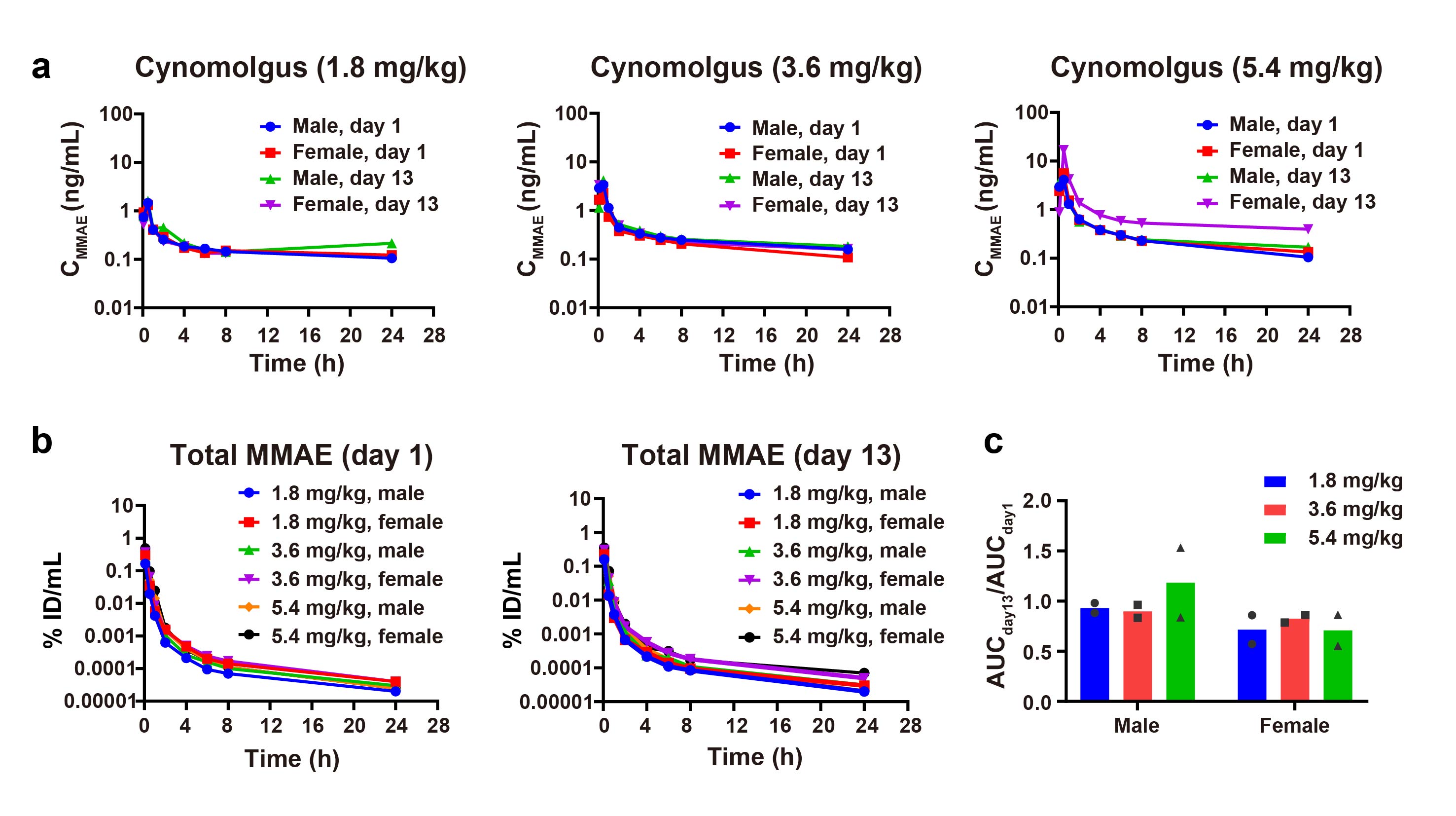


**Supplementary Fig. 21**

**TK study of Sgc8c-M in cynomolgus monkeys.** **(a)** Plasma concentration-time curve of free MMAE in cynomolgus monkeys after intravenous injection of Sgc8c-M at three doses (1.8, 3.6, and 5.4 mg/kg) on day 1 and day 13 during the course of the repeated-dose TK study. **(b)** % ID/mL of total MMAE on day 1 and day 13 in cynomolgus monkeys. **(c)** AUC ratio of total MMAE on day 13 and day 1 in cynomolgus monkeys. Data are presented as the mean of *n* = 2.


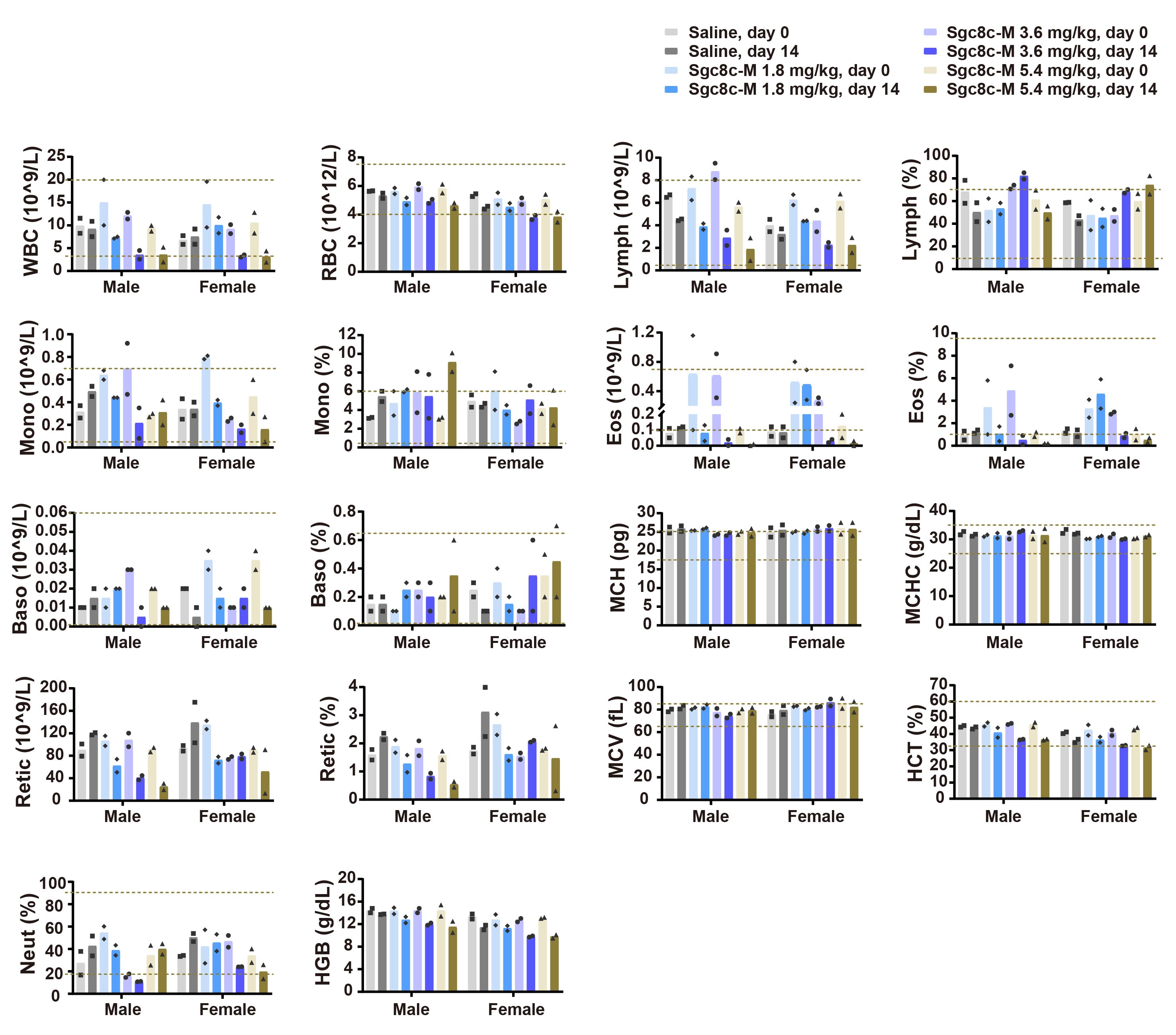


**Supplementary Fig. 22**

Blood routine parameters on day 0 (24 h before the first dose) and day 14 (24 h after the last dose) in the repeated-dose toxicity study of Sgc8c-M in cynomolgus monkeys. The results shown here are from the same experiment as that shown in Fig. 7d. Data are presented as the mean of *n* = 2.


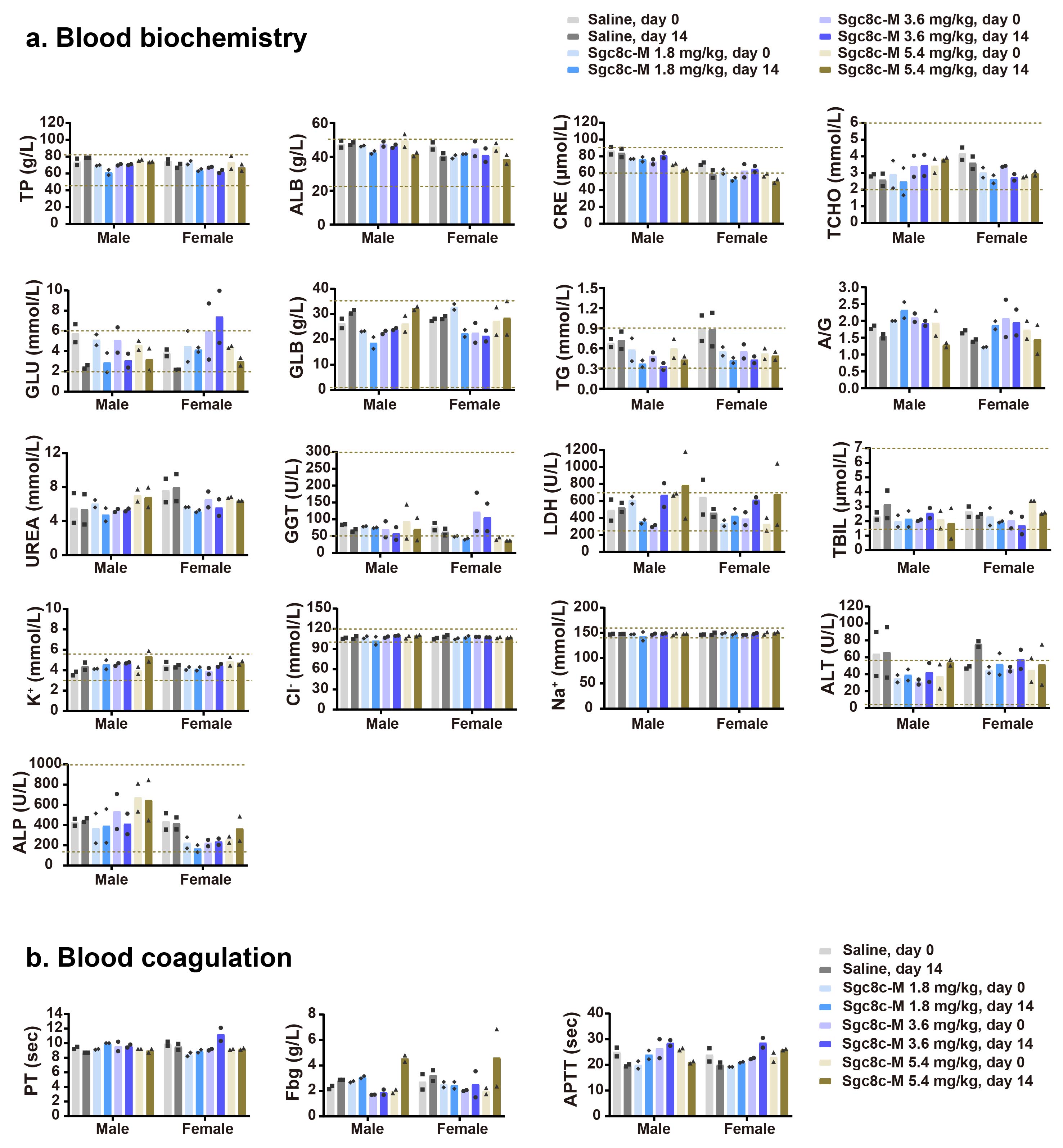


**Supplementary Fig. 23**

**(a)** Blood biochemical and **(b)** blood coagulation parameters on day 0 (24 h before the first dose) and day 14 (24 h after the last dose) in the repeated-dose toxicity study of Sgc8c-M in cynomolgus monkeys. The results shown here are from the same experiment as that shown in Fig. 7d. Data are presented as the mean of *n* = 2.


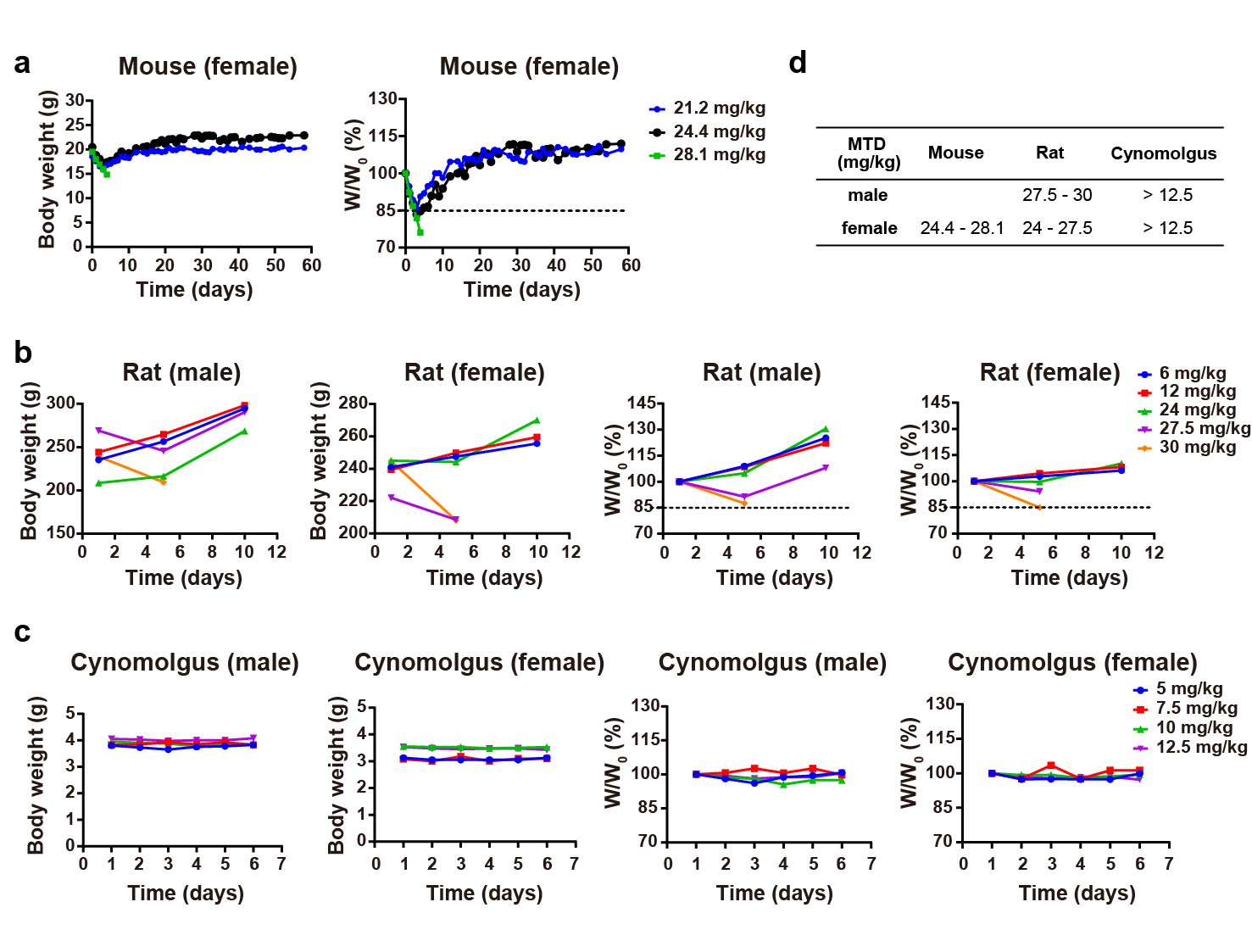


**Supplementary Fig. 24**

**Maximum tolerated dose (MTD) study of Sgc8c-M in mice, rats, and cynomolgus monkeys.** Changes in body weights and W/W_0_ (%) of BALB/c mice **(a)**, SD rats **(b)**, and cynomolgus monkeys **(c)** after single dosing of Sgc8c-M. **(d)** Summary of MTD values of Sgc8c-M in mice, rats, and cynomolgus monkeys. Data are presented as the mean of *n* = 2.


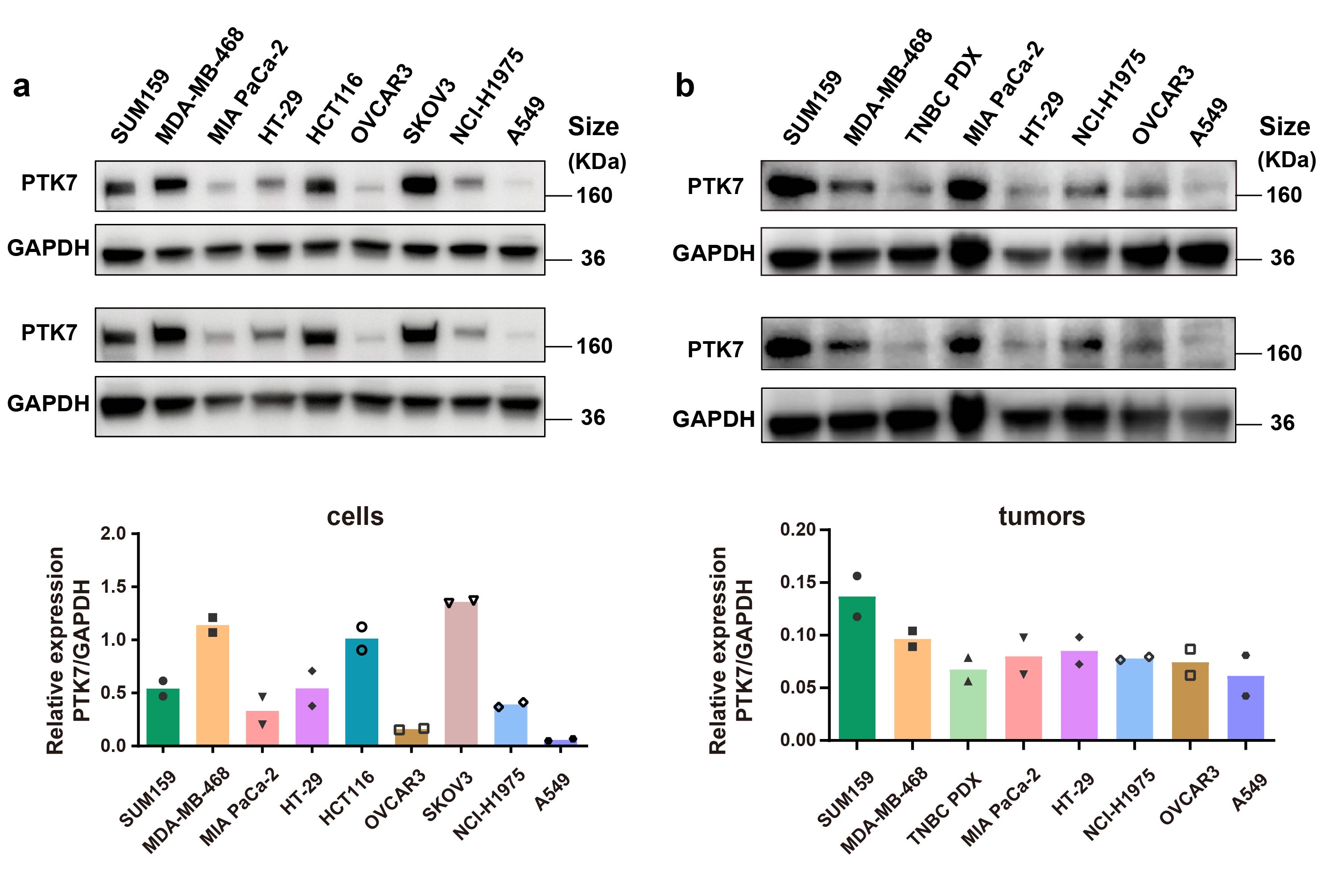


**Supplementary Fig. 25**

**Westen blot analysis of PTK7 expression in multiple cancer cells and tumors.** **(a)** Relative PTK7 expression in various cancer cells. **(b)** Relative PTK7 expression in various tumors.

| **Mice** | **PK Parameters** | **free MMAE** | | **total MMAE** | |
| --- | --- | --- | --- | --- | --- |
|  | Dose (mg/kg) | 7 | 14 | 7 | 14 |
|  | t_1/2_ (h) | 0.40 ± 0.05 | 0.90 ± 0.08 | 0.30 ± 0.02 | 0.63 ± 0.05 |
|  | T_max_ (h) | 0.08 ± 0.00 | 0.08 ± 0.00 | 0.08 ± 0.00 | 0.08 ± 0.00 |
|  | C_max_ (ng/ml) | 244.97 ± 22.33 | 471.42 ± 28.44 | 2078.94 ± 303.64 | 4972.14 ± 747.04 |
|  | C_0_ (ng/ml) | 315.11 ± 38.76 | 557.67 ± 37.55 | 2609.90 ± 460.44 | 6156.06 ± 916.13 |
|  | AUC_0-t_ (ng/ml*h) | 148.45 ± 9.24 | 336.64 ± 19.94 | 1165.95 ± 91.75 | 2814.19 ± 401.78 |
|  | AUC_0-inf_ (ng/ml*h) | 152.86 ± 8.06 | 341.34 ± 18.09 | 1175.54 ± 89.05 | 2818.70 ± 400.23 |
|  | MRT_0-inf_ (h) | 0.52 ± 0.03 | 0.75 ± 0.06 | 0.44 ± 0.04 | 0.44 ± 0.03 |
| **Rats** | **PK Parameters** | **free MMAE** | | **total MMAE** | |
|  | Dose (mg/kg) | 3.5 | 7.0 | 3.5 | 7.0 |
|  | t_1/2_ (h) | 9.05 ± 1.15 | 13.42 ± 3.48 | 8.84 ± 4.78 | 11.65 ± 8.02 |
|  | T_max_ (h) | 0.14 ± 0.10 | 0.08 ± 0.00 | 0.08 ± 0.00 | 0.08 ± 0.00 |
|  | C_max_ (ng/ml) | 13.08 ± 5.05 | 28.17 ± 6.13 | 1596.67 ± 28.87 | 3023.33 ± 433.17 |
|  | C_0_ (ng/ml) | 14.37 ± 7.88 | 37.14 ± 8.98 | 2553.43 ± 45.28 | 4647.17 ± 852.16 |
|  | AUC_0-t_ (ng/ml*h) | 15.36 ± 0.70 | 28.89 ± 5.30 | 646.60 ± 16.58 | 1425.52 ± 133.39 |
|  | AUC_0-inf_ (ng/ml*h) | 17.10 ± 0.86 | 35.29 ± 4.88 | 650.54 ± 12.59 | 1450.96 ± 116.99 |
|  | MRT_0-inf_ (h) | 7.94 ± 0.80 | 12.40 ± 3.53 | 0.82 ± 0.03 | 1.69 ± 0.73 |
| **Monkeys** | **PK Parameters** | **free MMAE** | | **total MMAE** | |
|  | Dose (mg/kg) | 1.8 | 3.6 | 1.8 | 3.6 |
|  | t_1/2_ (h) | 15.20 ± 2.50 | 53.35 ± 10.49 | 11.34 ± 0.93 | 12.85 ± 1.77 |
|  | T_max_ (h) | 0.14 ± 0.10 | 0.25 ± 0.00 | 0.08 ± 0.00 | 0.08 ± 0.00 |
|  | C_max_ (ng/ml) | 4.07 ± 0.94 | 10.37 ± 1.45 | 707.33 ± 100.72 | 1763.33 ± 275.02 |
|  | C_0_ (ng/ml) | 4.03 ± 1.12 | 7.15 ± 1.48 | 1275.36 ± 195.38 | 2685.87 ± 298.39 |
|  | AUC_0-t_ (ng/ml*h) | 4.93 ± 0.41 | 20.99 ± 1.55 | 229.77 ± 26.82 | 687.93 ± 112.32 |
|  | AUC_0-inf_ (ng/ml*h) | 5.25 ± 0.35 | 37.12 ± 2.26 | 230.42 ± 26.74 | 690.53 ± 112.16 |
|  | MRT_0-inf_ (h) | 12.82 ± 3.05 | 62.88 ± 13.21 | 0.92 ± 0.14 | 1.07 ± 0.09 |

**Supplementary Table 1**

Summary of calculated PK parameters of free and total MMAE in plasma after administration of Sgc8c-M to BALB/c nude mice, SD rats, and cynomolgus monkeys. Data are presented as the mean ± SD (*n* = 3). The short elimination half-life (t_1/2_) calculated in mice was due to the lack of long-time point data, which was attributed to the [restricted](javascript:;) limit of quantification (LOQ) of the analytical instrument used for LC-MS/MS quantification in our laboratory. The instrument is only capable of quantifying time points for a maximum of 2 hours (for 7 mg/kg Sgc8c-M) and 6 hours (for 14 mg/kg Sgc8c-M) following administration.

| **Rats** | **7 mg/kg Sgc8c-M** | | |
| --- | --- | --- | --- |
|  | AUC _heart_ | ng/g*h | 774.27 ± 25.62 |
|  | AUC _liver_ | ng/g*h | 1490.67 ± 169.88 |
|  | AUC _spleen_ | ng/g*h | 2547.33 ± 247.18 |
|  | AUC _lung_ | ng/g*h | 664.23 ± 135.98 |
|  | AUC _kidney_ | ng/g*h | 8343.33 ± 461.88 |
|  | AUC _large intestine_ | ng/g*h | 1332.40 ± 390.10 |
|  | AUC _small intestine_ | ng/g*h | 1008.63 ± 609.68 |
|  | AUC _stomach_ | ng/g*h | 742.07 ± 201.92 |
|  | AUC _bladder_ | ng/g*h | 1683.33 ± 630.25 |

**Supplementary Table 2**

AUC values of total MMAE in major tissues 24 h after administration of 7 mg/kg Sgc8c-M to SD rats. Data are presented as the mean ± SD (*n* = 3).

| **Rats** | **date** | **parameters** | **3.5 mg/kg** | | **10.5 mg/kg** | | **18 mg/kg** | |
| --- | --- | --- | --- | --- | --- | --- | --- | --- |
|  |  |  | **male** | **female** | **male** | **female** | **male** | **female** |
|  | **day 1** | t_1/2_ (h) | 8.03 ± 0.57 | 5.39 ± 0.81 | 6.96 ± 0.91 | 6.77 ± 1.75 | 9.25 ± 2.47 | 11.62 ± 0.76 |
|  |  | CL (mL/min/kg) | 5.59 ± 0.75 | 5.00 ± 0.58 | 3.52 ± 0.55 | 4.26 ± 1.04 | 3.56 ± 0.51 | 5.25 ± 0.65 |
|  |  | Vss (L/kg) | 0.25 ± 0.05 | 0.20 ± 0.02 | 0.13 ± 0.01 | 0.22 ± 0.10 | 0.19 ± 0.03 | 0.25 ± 0.02 |
|  |  | MRT_Inf_ (h) | 0.74 ± 0.07 | 0.69 ± 0.10 | 0.63 ± 0.05 | 0.81 ± 0.19 | 0.87 ± 0.09 | 0.80 ± 0.04 |
|  |  | AUC_last_ (h*ng/mL) | 529.15 ± 74.17 | 575.42 ± 76.50 | 2566.14 ± 348.71 | 2084.53 ± 406.87 | 4335.46 ± 751.29 | 2883.03 ± 344.98 |
|  |  | AUC_Inf_ (h*ng/mL) | 531.60 ± 74.04 | 576.37 ± 76.62 | 2572.33 ± 350.90 | 2091.10 ± 409.25 | 4360.03 ± 743.71 | 2905.49 ± 349.63 |
|  |  | AUC_Extrapolated_ (%) | 0.47 ± 0.09 | 0.17 ± 0.03 | 0.24 ± 0.05 | 0.31 ± 0.07 | 0.59 ± 0.31 | 0.77 ± 0.07 |
|  |  | C_0_ (ng/mL) | 2054.75 ± 259.34 | 2398.17 ± 286.45 | 8291.85 ± 997.71 | 6947.92 ± 2685.20 | 14062.64 ± 649.87 | 12962.42 ± 1503.89 |
|  | **day 13** | t_1/2_ (h) | 7.62 ± 0.12 | 6.33 ± 0.59 | 8.44 ± 0.55 | 9.99 ± 1.62 | 14.46 ± 7.83 | 9.52 ± 4.06 |
|  |  | CL (mL/min/kg) | 5.01 ± 0.39 | 6.28 ± 0.94 | 3.15 ± 0.33 | 4.36 ± 0.42 | 3.02 ± 1.45 | 5.65 ± 0.20 |
|  |  | Vss (L/kg) | 0.19 ± 0.02 | 0.23 ± 0.02 | 0.12 ± 0.01 | 0.22 ± 0.04 | 0.99 ± 0.57 | 0.40 ± 0.13 |
|  |  | MRT_Inf_ (h) | 0.62 ± 0.03 | 0.61 ± 0.07 | 0.64 ± 0.08 | 0.85 ± 0.09 | 7.35 ± 6.02 | 1.17 ± 0.39 |
|  |  | AUC_last_ (h*ng/mL) | 601.73 ± 53.55 | 478.77 ± 79.30 | 2815.23 ± 345.32 | 2040.19 ± 276.55 | 5078.55 ± 1817.18 | 2663.11 ± 111.39 |
|  |  | AUC_Inf_ (h*ng/mL) | 603.71 ± 53.71 | 480.06 ± 79.78 | 2824.80 ± 347.96 | 2055.93 ± 276.28 | 5860.45 ± 2653.41 | 2688.67 ± 95.49 |
|  |  | AUC_Extrapolated_ (%) | 0.33 ± 0.01 | 0.26 ± 0.07 | 0.33 ± 0.05 | 0.78 ± 0.13 | 10.41 ± 9.73 | 0.97 ± 0.64 |
|  |  | C_0_ (ng/mL) | 2681.03 ± 186.09 | 2298.67 ± 286.82 | 10316.87 ± 74.28 | 8505.69 ± 936.29 | 10309.80 ± 4038.38 | 9616.96 ± 1403.07 |

**Supplementary Table 3**

TK parameters of total MMAE in plasma after administration of Sgc8c-M to SD rats. Data are presented as the mean ± SD (*n* = 3).

| **Monkeys** | **date** | **parameters** | **1.8 mg/kg** | | **3.6 mg/kg** | | **5.4 mg/kg** | |
| --- | --- | --- | --- | --- | --- | --- | --- | --- |
|  |  |  | **male** | **female** | **male** | **female** | **male** | **female** |
|  | **day 1** | t_1/2_ (h) | 4.87 | 4.38 | 7.86 | 7.55 | 6.25 | 7.11 |
|  |  | CL (mL/min/kg) | 4.23 | 4.53 | 2.74 | 4.25 | 3.62 | 2.68 |
|  |  | Vss (L/kg) | 0.09 | 0.09 | 0.08 | 0.12 | 0.11 | 0.06 |
|  |  | MRT_Inf_ (h) | 0.38 | 0.37 | 0.46 | 0.49 | 0.53 | 0.38 |
|  |  | AUC_last_ (h*ng/mL) | 375.67 | 383.04 | 1127.68 | 724.07 | 1314.40 | 1740.16 |
|  |  | AUC_Inf_ (h*ng/mL) | 376.89 | 384.10 | 1131.08 | 726.20 | 1316.78 | 1742.39 |
|  |  | AUC_Extrapolated_ (%) | 0.33 | 0.29 | 0.30 | 0.30 | 0.20 | 0.13 |
|  |  | C_0_ (ng/mL) | 1431.31 | 1492.90 | 4276.17 | 2456.04 | 3589.86 | 5380.56 |
|  | **day 13** | t_1/2_ (h) | 4.11 | 5.06 | 6.85 | 7.42 | 6.21 | 8.41 |
|  |  | CL (mL/min/kg) | 4.61 | 6.12 | 3.04 | 5.19 | 3.14 | 3.84 |
|  |  | Vss (L/kg) | 0.09 | 0.13 | 0.09 | 0.18 | 0.08 | 0.15 |
|  |  | MRT_Inf_ (h) | 0.36 | 0.36 | 0.48 | 0.59 | 0.44 | 0.66 |
|  |  | AUC_last_ (h*ng/mL) | 353.42 | 254.78 | 1003.92 | 600.07 | 1462.13 | 1187.92 |
|  |  | AUC_Inf_ (h*ng/mL) | 354.31 | 255.74 | 1006.88 | 602.5 | 1464.44 | 1195.34 |
|  |  | AUC_Extrapolated_ (%) | 0.26 | 0.37 | 0.29 | 0.39 | 0.16 | 0.59 |
|  |  | C_0_ (ng/mL) | 1487.93 | 1178.27 | 3557.41 | 2103.51 | 4631.16 | 3712.00 |

**Supplementary Table 4**

TK parameters of total MMAE in plasma after administration of Sgc8c-M to cynomolgus monkeys. Data are presented as the mean (*n* = 2).

| **Flow rate** | **Time/min** | **A (0.1% FA H_2_O)** | **B (MeOH)** |
| --- | --- | --- | --- |
| 0.2 mL/min | 0 | 80% | 20% |
|  | 2 | 10% | 90% |
|  | 9 | 10% | 90% |

**Supplementary Table 5**

HPLC gradient condition for the separation of MMAE in LC-MS/MS.

| **Name** | **DNA sequence (5’-3’)** |
| --- | --- |
| Sgc8c | ATCTAACTGCTGCGCCGCCGGGAAAATACTGTACGGTTAGA |
| Sgc8c-SH | ATCTAACTGCTGCGCCGCCGGGAAAATACTGTACGGTTAGA-C6-SH |
| Ctrl-SH | AGATTGGCATGTCATAAAAGGGCCGCCGCGTCGTCAATCTA-C6-SH |
| Cy5-Sgc8c | Cy5-ATCTAACTGCTGCGCCGCCGGGAAAATACTGTACGGTTAGA |
| Cy5-Sgc8c-SH | Cy5-ATCTAACTGCTGCGCCGCCGGGAAAATACTGTACGGTTAGA-C6-SH |
| Cy5-Ctrl | Cy5-AGATTGGCATGTCATAAAAGGGCCGCCGCGTCGTCAATCTA |
| Cy5-Ctrl-SH | Cy5-AGATTGGCATGTCATAAAAGGGCCGCCGCGTCGTCAATCTA-C6-SH |

**Supplementary Table 6**

Names, chemical modifications, and sequences of DNA.


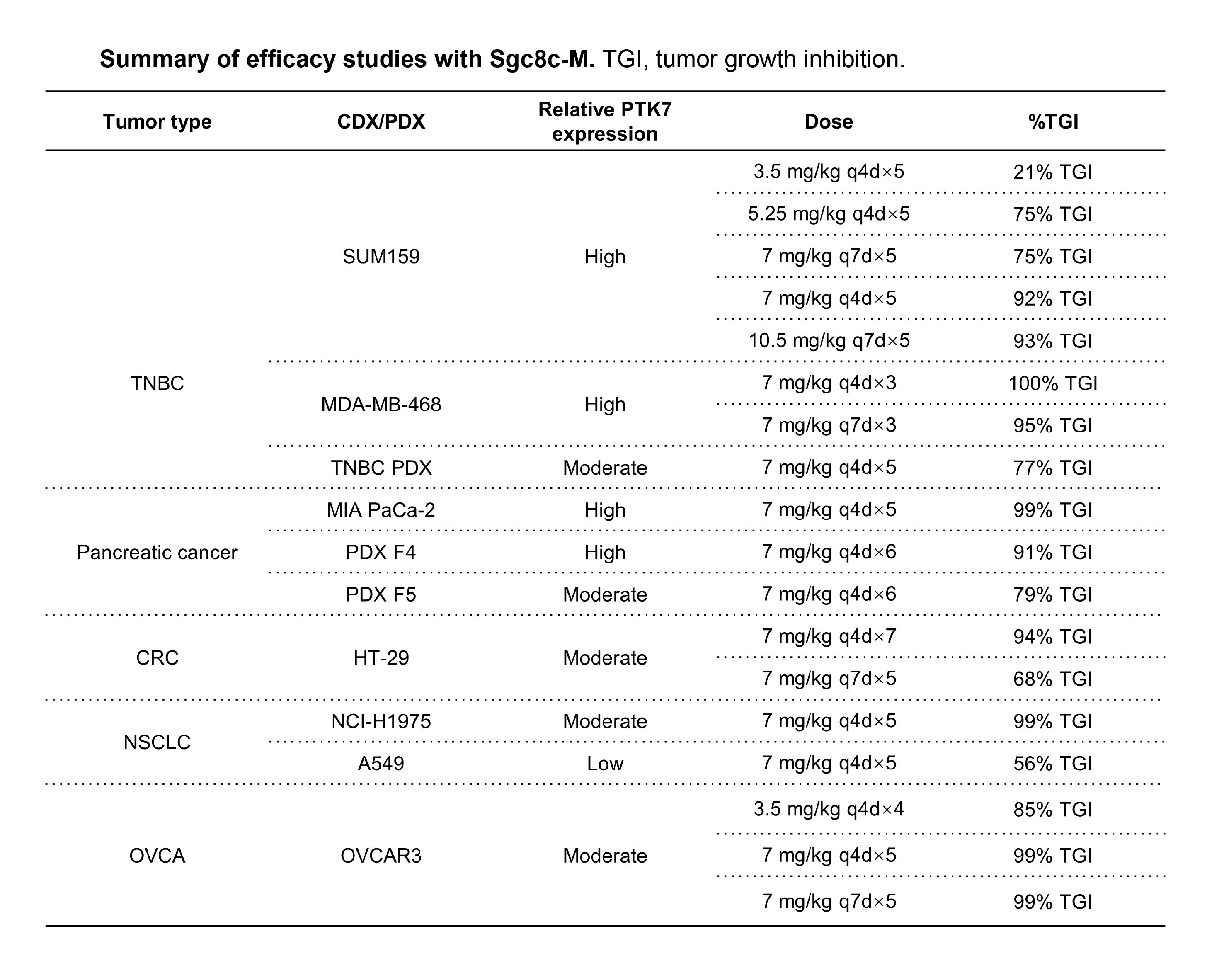


**Supplementary Table 7**

**Summary of efficacy studies with Sgc8c-M.** %TGI was calculated as follows: TGI (%) = [1 – (mean of tumor volume in the treatment group)/ (mean of tumor volume in the saline group)] × 100. The %TGI shown here is the calculated value on the day of the end of the observation period in the saline group.
